# The *Arabidopsis* Protein Phosphatase PP2C38 Negatively Regulates the Central Immune Kinase BIK1

**DOI:** 10.1371/journal.ppat.1005811

**Published:** 2016-08-05

**Authors:** Daniel Couto, Roda Niebergall, Xiangxiu Liang, Christoph A. Bücherl, Jan Sklenar, Alberto P. Macho, Vardis Ntoukakis, Paul Derbyshire, Denise Altenbach, Dan Maclean, Silke Robatzek, Joachim Uhrig, Frank Menke, Jian-Min Zhou, Cyril Zipfel

**Affiliations:** 1 The Sainsbury Laboratory, Norwich Research Park, Norwich, United Kingdom; 2 Institute of Genetics and Developmental Biology, Chinese Academy of Sciences, Beijing, China; 3 Max-Planck-Institute for Plant Breeding Research, Cologne, Germany; 4 Botanical Institute III, University of Cologne, Cologne, Germany; Texas A & M University, UNITED STATES

## Abstract

Plants recognize pathogen-associated molecular patterns (PAMPs) via cell surface-localized pattern recognition receptors (PRRs), leading to PRR-triggered immunity (PTI). The *Arabidopsis* cytoplasmic kinase BIK1 is a downstream substrate of several PRR complexes. How plant PTI is negatively regulated is not fully understood. Here, we identify the protein phosphatase PP2C38 as a negative regulator of BIK1 activity and BIK1-mediated immunity. PP2C38 dynamically associates with BIK1, as well as with the PRRs FLS2 and EFR, but not with the co-receptor BAK1. PP2C38 regulates PAMP-induced BIK1 phosphorylation and impairs the phosphorylation of the NADPH oxidase RBOHD by BIK1, leading to reduced oxidative burst and stomatal immunity. Upon PAMP perception, PP2C38 is phosphorylated on serine 77 and dissociates from the FLS2/EFR-BIK1 complexes, enabling full BIK1 activation. Together with our recent work on the control of BIK1 turnover, this study reveals another important regulatory mechanism of this central immune component.

## Author Summary

Plants use immune receptors at the cell surface to perceive microbial molecules and initiate a broad-spectrum defence response against pathogens. However, the induction and amplitude of immune signalling must be tightly regulated. Immune responses are triggered by ligand binding to a cognate receptor, which is present in dynamic kinase complexes that heavily rely on trans-phosphorylation to initiate signalling. The cytoplasmic kinase BIK1 associates with different immune receptors and plays a central role in the activation of downstream immune signalling. We show here that the *Arabidopsis thaliana* protein phosphatase PP2C38 negatively regulates immune responses by controlling the phosphorylation and activation status of BIK1. Furthermore, we propose a mechanism that relieves this negative regulation involving PP2C38 phosphorylation and dissociation from BIK1. These findings extend our knowledge on how plant immunity is appropriately regulated.

## Introduction

Recognition of pathogen-associated molecular patterns (PAMPs) by pattern recognition receptors (PRRs) initiates a complex signalling cascade leading to PRR-triggered immunity (PTI) [1, 2]. In plants, PRRs are plasma membrane (PM)-localized receptor kinases (RKs) or receptor-like proteins (RLPs) [3]. These PRRs typically form dynamic complexes with other regulatory RKs to initiate immune signalling [4, 5]. The *Arabidopsis thaliana* (hereafter *Arabidopsis*) leucine-rich repeat (LRR)-RKs FLS2 and EFR, which respectively recognize the bacterial PAMPs flagellin (or the epitope flg22) and EF-Tu (or the epitope elf18), are amongst the best-studied plant PRRs [6, 7]. Upon ligand binding, FLS2 and EFR rapidly form a heteromeric complex with the LRR-RK BAK1/SERK3 resulting in the phosphorylation of both RKs [8–13]. This initiates a series of downstream responses, such as changes in ion fluxes, reactive oxygen species (ROS) production, activation of mitogen-activated protein kinase (MAPK) cascades, transcriptional reprogramming, callose deposition, and finally immunity against microbial pathogens [14].

Recently, several mechanisms that regulate BAK1-mediated signalling in *Arabidopsis* prior or after PAMP perception have been identified. In the absence of the corresponding ligand, the formation of the PRR-BAK1 complex is prevented by the LRR-RK BIR2 [15], while BAK1 phosphostatus is controlled by a specific PP2A holoenzyme [16]. Following ligand binding, the BAK1-mediated interaction of the *Arabidopsis* E3-ubiquitin ligases PUB12 and PUB13 with FLS2 contributes to its degradation [17, 18], possibly to desensitize cells to flg22 stimuli.

In the resting state, FLS2 and EFR associate with the subfamily VII receptor-like cytoplasmic kinases (RLCK) BIK1 and related PBL proteins [19, 20]. Upon PAMP perception, the PRR-BAK1 complex directly phosphorylates BIK1 triggering its dissociation [19, 20]. BIK1 also associates with the LysM-RK CERK1 and the LRR-RK PEPR1, which mediate immune responses to fungal chitin and to the damage-associated molecular pattern (DAMP) AtPep1 (and related AtPep peptides), respectively [20, 21]. Thus, BIK1 has emerged as a central and convergent regulator of distinct PRR-dependent pathways playing a key positive role in PTI responses, such as the generation of ROS and Ca^2+^ bursts, and induced resistance to pathogens [20, 22–26]. Notably, upon PAMP perception BIK1 directly phosphorylates the NADPH oxidase RBOHD to activate ROS production, which is crucial for triggering PAMP-induced stomatal closure, an early PTI response thought to restrict pathogen entry into leaf tissues [23, 24]. Additionally, RBOH enzymes are positively regulated through direct Ca^2+^ binding to conserved EF-hand motifs and via Ca^2+^-dependent protein kinase (CDPK)-mediated phosphorylation [27–29]. BIK1-mediated RBOHD phosphorylation has been proposed to prime RBOHD for the subsequent Ca^2+^-dependent regulation [30]. Accordingly, loss of BIK1 or BIK1-mediated phosphorylation of RBOHD severely compromises ROS production, resulting in deficient stomatal immunity against hypovirulent *Pseudomonas syringae* strains [23, 24]. The biological importance of BIK1 (and related PBL proteins) is further demonstrated by the fact that bacteria, such as *P*. *syringae* and *Xanthomonas campestris*, secrete type-III secreted effectors into plant cells to cleave or inhibit these kinases, and thus block their action [20, 31].

It is becoming increasingly clear that the first immediate downstream substrates of activated RKs complexes at the PM are RLCKs. This is particularly evident in PTI signalling with the important role of BIK1 and related PBL proteins acting downstream of FLS2, EFR and PEPR1 [19–21]. In addition, the related RLCKs OsRLCK185 and PBL27 regulate chitin signalling downstream of CERK1 in rice and *Arabidopsis*, respectively [32, 33], while BSK1 dynamically associates with FLS2 to regulate flg22-induced responses [34]. Recently, the RLCK PCRK1 was also shown to contribute to FLS2 signalling [35], although it is still unknown whether PCRK1 interacts with FLS2. Despite the importance of RLCKs for RK-mediated signalling, particularly as links between activated PRRs and downstream immune outputs, hardly anything is known about the negative regulation of RLCKs. BIK1 protein accumulation is controlled by the CDPK CPK28 in a 26S proteasome-dependent manner, in order to modulate the amplitude of PTI signalling [36]. Interestingly, the heterotrimeric G proteins XLG2, AGB1 and AGG1/AGG2 were recently shown to attenuate proteasomal BIK1 degradation [37], although it is unclear whether these G proteins act by regulating CPK28-mediated BIK1 degradation. Crucially, CPK28 does not affect PAMP-induced BIK1 hyper-phosphorylation, suggesting that additional regulatory mechanisms exist.

Here, we describe the role of the previously uncharacterized *Arabidopsis* protein phosphatase PP2C38 in the regulation of BIK1 activation. We followed biochemical and genetic approaches to show that PP2C38 negatively regulates BIK1-mediated immune responses by controlling BIK1 phosphorylation and activation status. Notably, PAMP perception leads to PP2C38 phosphorylation and release from BIK1, presumably to enable full BIK1 activation. Our work reveals a novel mechanism of immune signalling regulation through the control of BIK1 phosphorylation status, while providing an example of a protein phosphatase targeting an RLCK in plants.

## Results

### PP2C38 associates dynamically with the EFR-BIK1 and FLS2-BIK1 complexes

To identify novel regulators of PRR complexes in *Arabidopsis*, we performed a yeast two-hybrid (Y2H) screen using the cytoplasmic domain of EFR as bait against a prey library generated from *Arabidopsis* cDNA. Given the crucial role of protein phosphorylation for activation of the PRR complex following PAMP perception and initiation of PTI signalling [4], we were particularly interested in two PP2C-type protein phosphatases, PP2C38 (At3g12620: [38]; also named PP2C-D3 [39] or APD1 [40]) and PP2C58 (At4g28400), retrieved from this initial screen (S1 Table).

**Fig 1:**
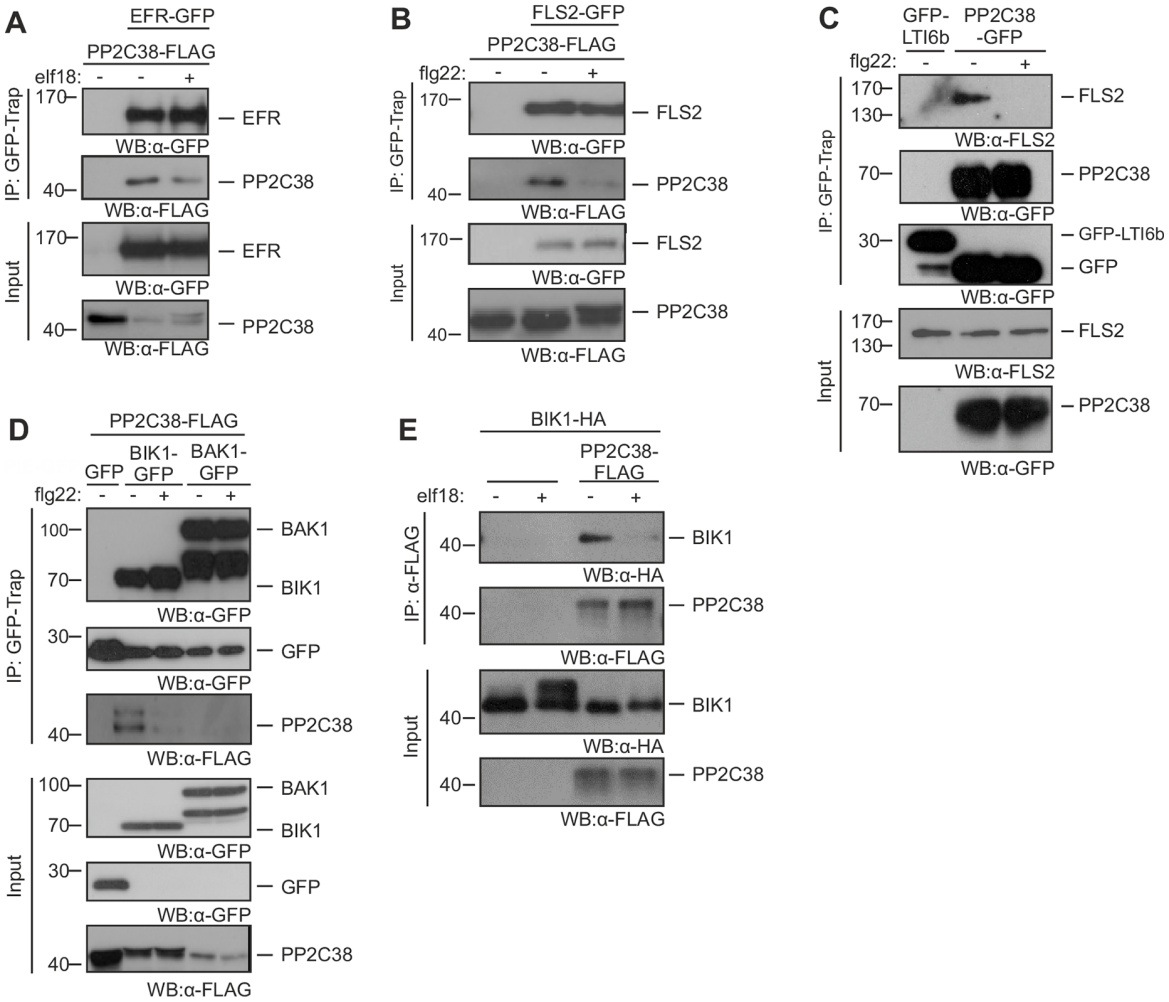
PP2C38 associates dynamically with the EFR-BIK1 and FLS2-BIK1 complexes. (A-B) Co-immunoprecipitation of PP2C38 and EFR (A) or FLS2 (B) transiently expressed in *N*. *benthamiana* leaves treated (+) or not (-) with 100 nM elf18 (A) or flg22 (B) for 20 min. (C) Co-immunoprecipitation of PP2C38 and FLS2 in stable transgenic *Arabidopsis* seedlings (T3) expressing PP2C38-GFP. Seedlings were treated (+) or not (-) with 1 μM flg22 for 20 min. Native FLS2 protein visualized in immunoblot using α-FLS2 antibody. The PM marker GFP-LTI6b [71] was used as a negative control. (D) Co-immunoprecipitation of PP2C38 and BIK1 or BAK1 transiently expressed in *N*. *benthamiana* leaves treated (+) or not (-) with 100 nM flg22 for 20 min. (E) Co-immunoprecipitation of PP2C38 and BIK1 transiently expressed in *Arabidopsis* Col-0 protoplasts. Protoplasts were treated (+) or not (-) with 1 μM elf18 for 30 min. All experiments were performed at least three times with similar results.

To test if PP2C38 and PP2C58 associate with EFR *in planta*, we transiently co-expressed full-length EFR-GFP with PP2C38-FLAG or PP2C58-FLAG in *Nicotiana benthamiana*. After immunoprecipitation using GFP-Trap beads we detected a specific association between EFR-GFP and PP2C38-FLAG (Fig 1A). However, the association between EFR-GFP and PP2C58-FLAG appeared nonspecific as PP2C58-FLAG also co-immunoprecipitated with free GFP (S1A Fig). Similarly, no association could be detected between PP2C58-HA and EFR-GFP (S1B Fig). Hence, we decided to focus our study on the biochemical and functional characterization of PP2C38.

Remarkably, we consistently observed reduced levels of co-immunoprecipitated PP2C38-FLAG (but not of total protein) after elf18 treatment (Fig 1A), indicating that PP2C38 dissociates from EFR after elf18 perception. Given the commonality of signalling components between the FLS2 and EFR pathways [4], we tested whether PP2C38 also associates *in planta* with FLS2. As observed with EFR, PP2C38-FLAG formed a complex with FLS2-GFP, which was disrupted after flg22 treatment (Fig 1B). Intriguingly, we noted that both elf18 and flg22 treatment induced a band shift of PP2C38-FLAG protein on the immunoblot (Fig 1A and 1B). To confirm the observed associations in *Arabidopsis*, we generated a homozygous transgenic line expressing *PP2C38-GFP* (Fig 1C and S2 Fig). Using this line, we detected endogenous FLS2 in PP2C38-GFP immunoprecipitates in mock-treated but not in flg22-treated seedlings (Fig 1C). Taken together, we conclude that PP2C38 forms a complex with FLS2 and EFR, and that this association is destabilized upon ligand perception.

EFR and FLS2 form dynamic complexes with different kinases, such as BAK1 and BIK1 [8, 12, 13, 19, 20]. Therefore, we tested whether PP2C38 also associates with these kinases. Co-immunoprecipitation of transiently expressed epitope-tagged proteins in *N*. *benthamiana* showed that PP2C38-FLAG also associates with BIK1-GFP (Fig 1D). Interestingly, we noted that PP2C38-FLAG exhibits a constitutive band shift when co-expressed with BIK1-GFP (Fig 1D and S3 Fig). Similar to our previous observations with EFR and FLS2, PP2C38-FLAG dissociated from BIK1-GFP after flg22 treatment in *N*. *benthamiana* (Fig 1D). A similar observation was made after elf18 treatment in *Arabidopsis* protoplasts co-expressing PP2C38-FLAG and BIK1-HA (Fig 1E). In contrast, no association was detected between PP2C38-FLAG and BAK1-GFP in *N*. *benthamiana* (Fig 1D). Since C-terminally tagged BAK1 proteins are impaired in PTI signalling but not in their ligand-induced association with FLS2 [41], we tested if PP2C38-FLAG could associate with endogenous BAK1 in *Arabidopsis* protoplasts. In line with previous experiments (Fig 1D), we did not find evidence for their association (S4 Fig). Taken together, our results indicate that PP2C38 associates dynamically with BIK1, in addition to forming a dynamic complex with EFR and FLS2.

### PP2C38 is an active PM-localized phosphatase

**Fig 2:**
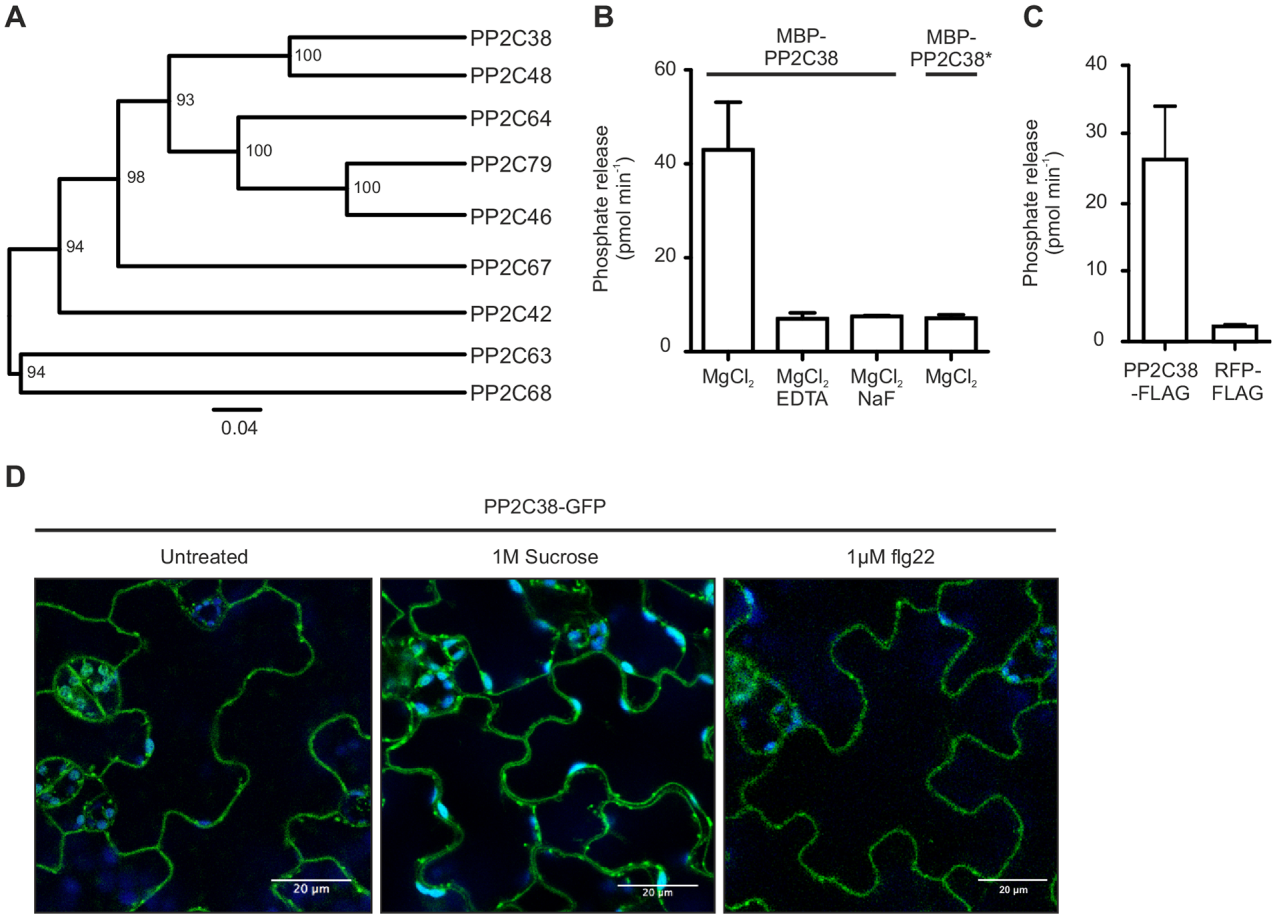
PP2C38 is an active plasma membrane-localized phosphatase. (A) Phylogenetic analysis of *Arabidopsis* PP2C clade D. Protein sequences were aligned using clustalW and the tree generated using UPGMA. Numbers indicate bootstrap values from 100 replicates. (B) PP2C38 is an active phosphatase *in vitro*. Recombinant MBP-PP2C38 or MBP-PP2C38* proteins (where PP2C38* is a catalytically-inactive variant) were incubated with a synthetic phosphopeptide in the presence or absence of Mg^2+^ ions, cation chelator EDTA or phosphatase inhibitor NaF. Release of inorganic phosphate was quantified using a colorimetric assay. Values are averages ± SD (n = 3). (C) PP2C38 is an active phosphatase *in vivo*. Immunoprecipitated PP2C38-FLAG or control RFP-FLAG proteins from *N*. *benthamiana* leaves were incubated with a synthetic phosphopeptide, and release of inorganic phosphate was quantified using a colorimetric assay. (D) PP2C38 localizes to the plasma membrane. Confocal microscopy of *Arabidopsis* cotyledons stably expressing *PP2C38-GFP*. Plasmolysis (arrows) with 1M sucrose indicates plasma membrane localization. Treatment with 1 μM flg22 for 30 min did not alter PP2C38 localization.

PP2C38 belongs to the clade D of *Arabidopsis* PP2Cs together with eight other members (Fig 2A) [42]. A BLAST analysis on the available genomes of several plant species retrieved numerous PP2C38 orthologs, including in monocots (S5 Fig). To test if PP2C38 is a catalytically active protein phosphatase, we incubated recombinant MBP-PP2C38 with a generic synthetic phosphopeptide and assessed the release of inorganic phosphate in a colorimetric assay. PP2C38 exhibited typical Mg^2+^-dependent PP2C activity (Fig 2B). As a control, we generated a phosphatase-inactive PP2C38* variant by converting two aspartic acids (D87 and D289) that are required for the coordination of Mg^2+^ ions during catalysis into asparagine [43]. As expected, this variant was completely devoid of catalytic activity (Fig 2B). Similarly, we detected phosphatase activity from transiently expressed PP2C38-FLAG protein purified from *N*. *benthamiana* leaves (Fig 2C).

To gain insight into PP2C38 function, we determined its subcellular localization by confocal microscopy analysis of cotyledons of *Arabidopsis* seedlings stably expressing *PP2C38-GFP*. We detected a strong GFP signal at the PM and in intracellular punctea (Fig 2D). The PM localization was further confirmed by induction of plasmolysis with a hyperosmotic sucrose solution (Fig 2D). No change in localization was observed upon flg22 treatment (Fig 2D). PP2C38 has a predicted palmitoylation site at position C154 (according to CSS-Palm prediction software [44]), which may explain its PM localization. Thus, while the observed sub-cellular localization pattern would need to be confirmed upon native expression (NB: we have never managed to detect PP2C38-GFP by confocal microscopy when expressed under its native promoter), the observed PM localization of PP2C38 is consistent with its interaction with EFR, FLS2 and the PM-associated cytoplasmic kinase BIK1 (Fig 1), and suggests a potential role of PP2C38 in the regulation of PRR complexes.

### PP2C38 regulates BIK1 phosphostatus and activity

**Fig 3:**
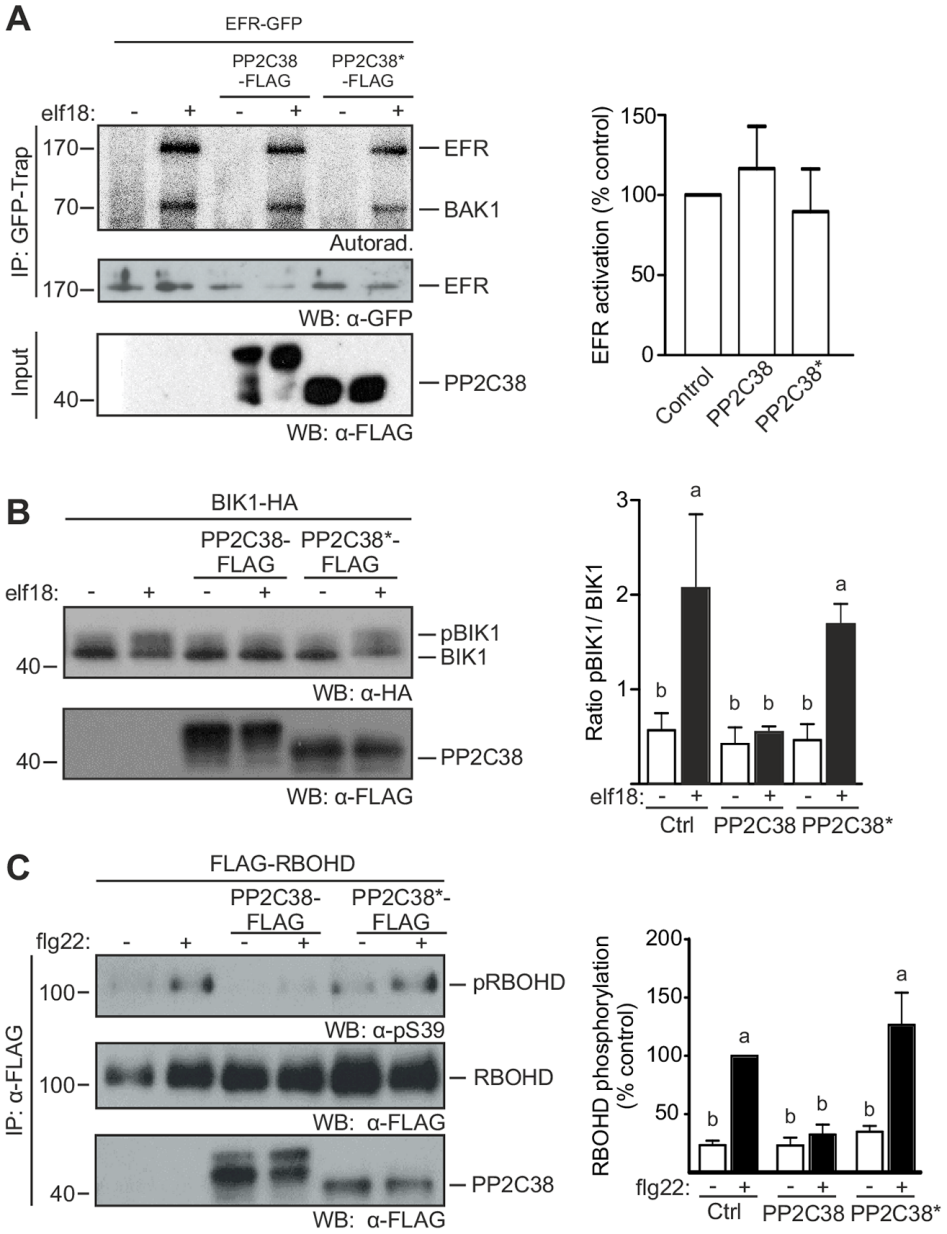
PP2C38 regulates BIK1 activation. (A) PP2C38 does not affect EFR activation. Protoplasts from *efr/pEFR*:*EFR-GFP* transgenic *Arabidopsis* plants transfected with PP2C38/PP2C38*-FLAG were treated with water (-) or 1 μM elf18 (+). Immunoprecipitated EFR-GFP was incubated with [^32^P]γ-ATP. *In vitro* phosphorylation is revealed by autoradiography. Graph (right panel) represents the densitometry measurements from three independent experiments. (B) PP2C38 negatively regulates elf18-induced BIK1 hyper-phosphorylation. *Arabidopsis* Col-0 protoplasts co-transfected with BIK1-HA and PP2C38/PP2C38*-FLAG were treated with water (-) or 1 μM elf18 (+).BIK1 phosphorylation ratio (right panel) calculated using densitometry measurements from three independent experiments; values are mean ± SE. Letters indicate significantly different values at *p* < 0.05 based on one-way ANOVA, Dunnett’s comparison test to control samples. (C) PP2C38 inhibits elf18-triggered BIK1-dependent RBOHD phosphorylation. *Arabidopsis* Col-0 protoplasts co-transfected with FLAG-RBOHD and PP2C38/PP2C38*-FLAG were treated with water (-) or 1 μM flg22 (+). FLAG-RBOHD proteins were immunoprecipitated and S39 phosphorylation was analysed using α-pS39 antibodies. Phosphorylation ratio (right panel) calculated using densitometry measurements from pRBOHD and immunoprecipitated RBOHD immunoblots, normalized to control (treated) samples. Values are mean ± SE from 4 independent experiments. Letters indicate significantly different values at *p* < 0.01 based on one-way ANOVA, Dunnett’s comparison test to control samples.

EFR phosphorylation is greatly enhanced after ligand perception, a step that is crucial for the activation of EFR kinase [3, 9, 45]. Given the interaction between PP2C38 and EFR (Fig 1), we first tested whether PP2C38 over-expression affects elf18-induced EFR kinase activation. We transfected PP2C38-FLAG or PP2C38*-FLAG into protoplasts from *Arabidopsis* plants stably expressing EFR-GFP, and subsequently performed an *in vitro* kinase assay using ^32^P-radiolabelled ATP on immunoprecipitated EFR (IP-kinase assay). Phosphorylation of EFR was specifically detected after elf18 treatment (Fig 3A). Interestingly, this phosphorylation pattern was not affected by PP2C38 or PP2C38* over-expression (Fig 3A). Although PP2C38 associates with EFR (S1 Table; Fig 1A), we concluded that PP2C38 is most likely not involved in the regulation of EFR phosphorylation status. Also, consistent with the lack of evidence for PP2C38-BAK1 association (Fig 1D, S3 Fig), PP2C38 or PP2C38* over-expression did not affect the phosphorylation status of EFR-associated BAK1 upon elf18 treatment (Fig 3A).

Besides EFR and FLS2 (Fig 1A–1C), PP2C38 also associates with BIK1 (Fig 1D and 1E). To test if PP2C38 regulates BIK1 phosphorylation, we assessed the band shift of BIK1 induced by PAMP treatment due to hyper-phosphorylation [19, 20, 22]. Although, we often noted that BIK1 shows a constitutive band shift in Arabidopsis protoplasts, which may be due to the release of DAMP during their preparation, elf18 treatment could still enhance BIK1 phosphorylation (pBIK1) in *Arabidopsis* protoplasts expressing BIK1-HA (Fig 3B). BIK1 phosphorylation was markedly reduced when PP2C38-FLAG was co-transfected (Fig 3B). Importantly, the reduction of BIK1 phosphorylation specifically required PP2C38 phosphatase activity, as co-expression of PP2C38*-FLAG did not affect elf18-induced BIK1 phosphorylation (Fig 3B). Considering that PP2C38 over-expression did not alter EFR or BAK1 phosphorylation (Fig 3A), these results are consistent with the hypothesis that PP2C38 directly dephosphorylates BIK1.

Next, we investigated whether PP2C38-mediated inhibition of BIK1 hyper-phosphorylation affects its ability to phosphorylate downstream targets. We previously described the NADPH oxidase RBOHD as a direct substrate of BIK1 [23, 24]. We identified a number of BIK1-dependent RBOHD phosphosites [23, 24], which can be monitored using phosphosite-specific antibodies, and therefore be used as an effective proxy for BIK1 activation *in vivo*. We co-transfected FLAG-RBOHD with or without PP2C38/PP2C38*-FLAG in *Arabidopsis* protoplasts. FLAG-RBOHD was enriched by immunoprecipitation and phosphorylation of the BIK1-specific phosphosite S39 was assessed using anti-phospho S39 (α-pS39) antibodies [24]. After elf18 treatment, we detected a significant increase in RBOHD-S39 phosphorylation, which was significantly reduced when PP2C38-FLAG, but not PP2C38*, was co-expressed (Fig 3C). This clearly demonstrates the requirement of PP2C38 phosphatase activity for repressing BIK1-mediated RBOHD phosphorylation. Taken together, our results indicate that PP2C38 is a negative regulator of BIK1 phosphorylation status and activity.

### PP2C38 negatively regulates PAMP-induced ROS burst and stomatal immunity

**Fig 4:**
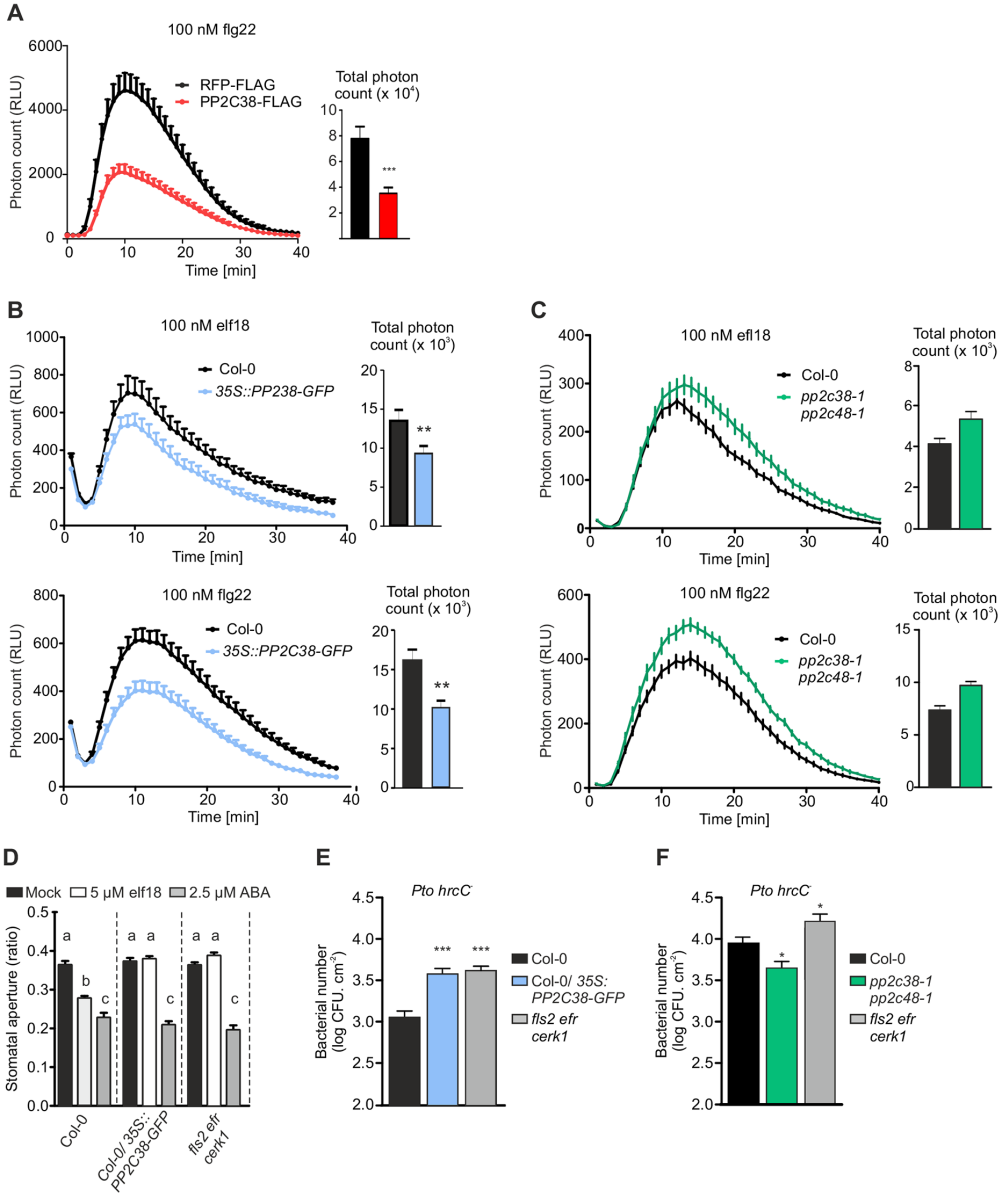
PP2C38 regulates PAMP-induced ROS burst and stomatal immunity. (A-B) Overexpression of *PP2C38* impairs the ROS burst induced by 100 nM flg22 in *N*. *benthamiana* (A), and by 100 nM flg22 or elf18 in *Arabidopsis* (B). Values are mean ± SE (n = 12) and are expressed in relative light units (RLU). Significant differences are designated by asterisks (***p < 0.001; **p < 0.01) based on unpaired Student’s t test. Experiment replicated three times with similar results. (C) Loss of PP2C38 and PP2C48 enhances ROS production induced by 100 nM elf18 or flg22. Values are mean ± SE (n ≥ 20) and are expressed in relative light units (RLU). Significant differences are designated by asterisks *p < 0.05) based on unpaired Student’s t test. Experiment replicated three times with similar results. (D) PP2C38 regulates elf18- but not ABA-induced stomatal closure. Stomatal aperture was measured 2 h after 5 μM elf18 or 2.5 μM ABA treatment. Values are mean ± SE (n>50; one-way ANOVA, Tukey post hoc test). Different letters indicate significantly different values at *p* < 0.001. Experiment replicated three times with similar results. (E-F) PP2C38 is a negative regulator of anti-bacterial immunity. *Pto* DC3000 *hrcC* ^*-*^ was sprayed onto leaf surface. Bacterial growth was determined 4 days post-inoculation. Values are mean ± SE (n = 4). Graph represents the combined results of three independent experiments. Asterisks indicate significant differences compared with Col-0 (one-way ANOVA, Dunnet post hoc test, *p < 0.05, ***p < 0.001). Cfu indicates colony-forming units.

We next sought to investigate the biological role of PP2C38-mediated BIK1 dephosphorylation. Having shown that PP2C38 inhibits PAMP-induced BIK1 hyper-phosphorylation and subsequent trans-phosphorylation of the NADPH oxidase RBOHD (Fig 3), we tested whether this translates into an inhibition of BIK1-mediated immune outputs. We measured the flg22-induced ROS burst in *N*. *benthamiana* after transient over-expression of PP2C38. We observed that leaves expressing PP2C38-FLAG exhibited significantly reduced flg22-induced ROS burst (Fig 4A). Similarly, stable homozygous *Arabidopsis* transgenic plants over-expressing *PP2C38-GFP* exhibited a significantly reduced flg22- and elf18-triggered ROS burst (Fig 4B). The negative impact of PP2C38 on PAMP-induced ROS burst was further confirmed using two additional independent homozygous lines over-expressing *PP2C38-GFP* in the *pp2c38-1* background (S6 Fig).

In addition, we tested whether loss of *PP2C38* expression would result in increased PAMP-induced ROS burst. Null single mutant plants (S2 Fig) mostly behave like wild-type plants in response to flg22 or elf18 treatment (S7 Fig). We therefore hypothesised that PP2C48, which shares over 90% sequence similarity with PP2C38 and is its closest homologue within the PP2C clade D (Fig 2A), may be functionally redundant with PP2C38. Thus, we also isolated an insertional mutant line for *PP2C48* (S2 Fig),generated a double *pp2c38-1 pp2c48-1* null mutant, and tested these plants for PAMP-induced ROS production. We consistently observed a small but significant increase in ROS production for *pp2c38-1 pp2c48-1* double mutants in response to elf18 and flg22 (a representative experiment is shown in Fig 4C and a comparison of different independent biological experiments applying a linear mixed effect model is shown in S7 Fig. A higher variability was observed for the *pp2c38-1* and *pp2c48-1* single mutants, which in some occasions exhibited an enhanced ROS burst (S7 Fig). While elf18-induced ROS burst was mostly unaffected in the single mutants, simultaneous analysis of several experiments revealed that *pp2c48-1* single mutants showed an increase in flg22-induced ROS burst comparable to that observed in the double mutant (S7 Fig). Together, our data indicate that PP2C38 and PP2C48 most likely act redundantly to negatively regulate PAMP-induced ROS burst, while the specific contributions of the respective phosphatases seem to differ depending on the eliciting PAMP.

PAMP-induced stomatal closure helps to prevent pathogens from entering leaf tissues [46]. This immune response is dependent on RBOHD and its activation by BIK1-mediated phosphorylation [20, 23, 24, 47, 48]. Loss of BIK1 activation or BIK1-mediated RBOHD phosphorylation leads to increased susceptibility to bacteria [22–24]. Consistent with the suggested role of PP2C38 in BIK1 dephosphorylation, we observed that elf18-induced stomatal closure was abolished in *PP2C38-GFP* over-expressing plants to the same extent as in the elf18-insensitive mutant line *fls2 efr cerk1* (Fig 4D). Similar results were obtained after treatment with flg22 (S8 Fig). Importantly, plants over-expressing *PP2C38-GFP* showed normal stomatal closure in response to abscisic acid (ABA) treatment (Fig 4D), demonstrating that the general stomatal closure machinery is not affected by *PP2C38* over-expression.

In accordance with these results, *PP2C38-GFP* over-expressing plants exhibited enhanced susceptibility to the non-pathogenic bacterial strain *P*. *syringae* pv. *tomato (Pto)* DC3000 *hrcC* ^*-*^ when compared to wild-type plants (Fig 4E). In contrast, *pp2c38-1 pp2c48-1* double mutant plants were slightly more resistant to *Pto hrcC* ^*-*^ (Fig 4F), which was consistent with their enhanced ROS production.

Together, our results support a model in which PP2C38 (and possibly PP2C48)-mediated BIK1 dephosphorylation constitutes an important regulatory mechanism for controlling stomatal immunity.

### Phosphorylation on serine 77 is required for the dissociation of PP2C38 from BIK1

**Fig 5:**
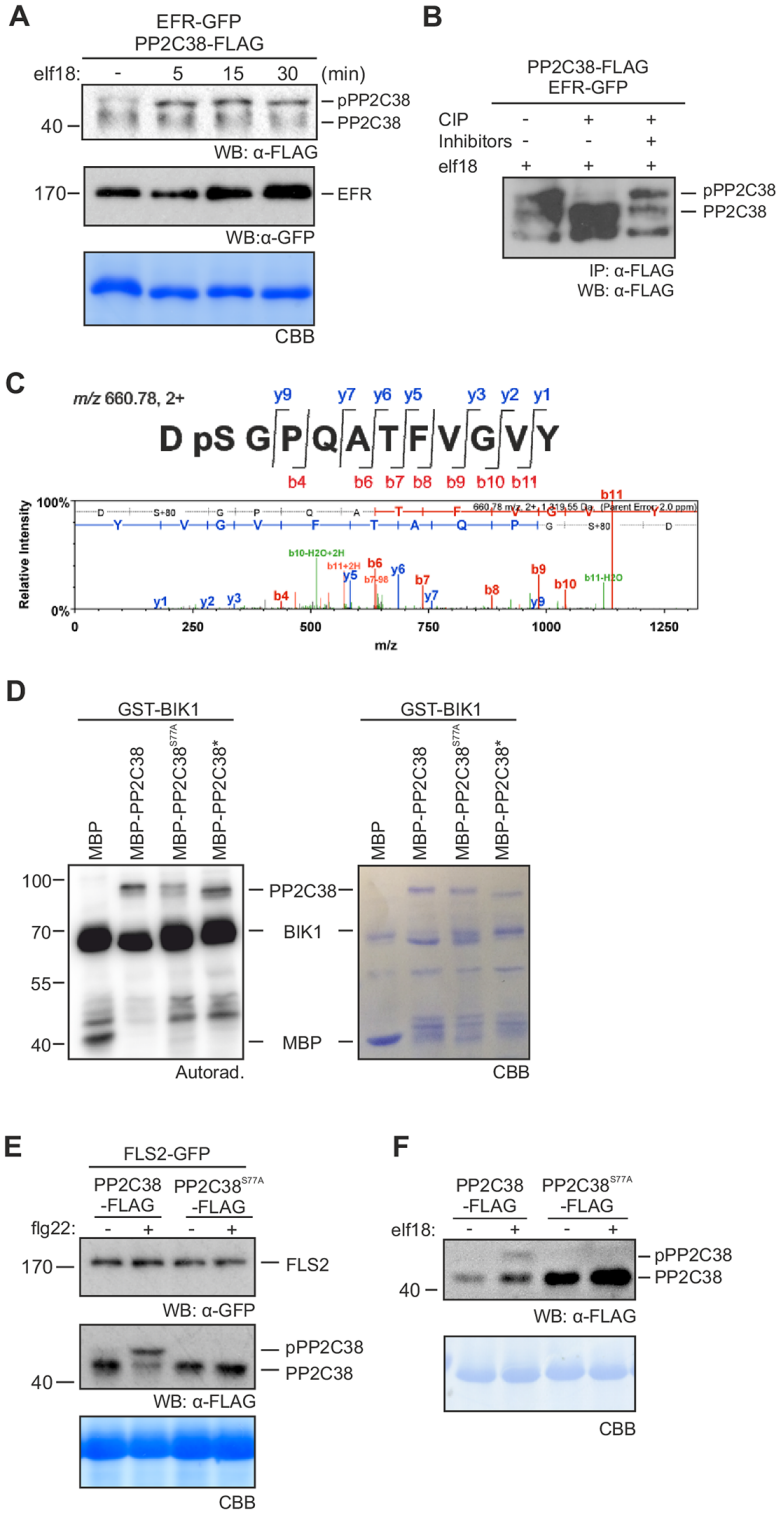
PP2C38 is phosphorylated in response to PAMP perception. (A) PAMP perception induces PP2C38 band shift. PPC2C38-FLAG protein expressed in *N*. *benthamiana* leaves and treated (+) or not (-) with 100 nM elf18 for the indicated times. Upper band corresponds to phosphorylated PP2C38 form (pPP2C38). 12% bisacrylamide gels were used for better protein separation. Experiment repeated two times with similar results. (B) PP2C band shift is due to phosphorylation. Immunoprecipitated PP2C38-FLAG proteins from *N*. *benthamiana* leaves co-expressing EFR-GFP treated with 100 nM elf18 were incubated with calf intestine phosphatase (CIP) in the presence or absence of the phosphatase inhibitor NaF. (C) PP2C38 is phosphorylated on S77 *in vivo*. Immunoprecipitated PP2C38-FLAG proteins from *N*. *benthamiana* were submitted for LTQ-Orbitrap MS/MS analysis. The DpSGPQATFVGVY phosphopeptide was identified as a doubly charged precursor (m/z 660.78) with fragmentation pattern consisting of singly and doubly charged b- and y- ions. Modified peptide sequence and fragmentation pattern shown above spectrum. (D) S77 phosphorylation is required for BIK1 trans-phosphorylation of PP2C38 *in vitro*. Recombinant GST-BIK1 was incubated with [^32^P]γ-ATP to promote auto-phosphorylation, followed by addition of recombinant MBP-PP2C38. *In vitro* PP2C38 trans-phosphorylation is revealed by autoradiography. CBB staining shown as loading control. Experiment repeated two times with similar results. (E-F) S77 is required for flg22-induced PP2C38 band shift. Phospho-dead PP2C38^S77A^-FLAG variant transiently expressed in *N*. *benthamiana* (E) or in *Arabidopsis* Col-0 protoplasts (F) does not exhibit a band shift after 20 min 100 nM flg22 treatment. 12% bisacrylamide gels were used for better protein separation. Experiments repeated at least three times with similar results.

We observed previously that PP2C38 dissociates from the BIK1 complex upon PAMP perception (Fig 1D and 1E), suggesting an active PAMP-induced mechanism to relieve PP2C38-mediated negative regulation. In addition, PP2C38-FLAG exhibited a band shift on SDS-PAGE after PAMP treatment (Fig 1A and 1B). In a more detailed time-course analysis, we detected a double band for PP2C38-FLAG already 5 minutes after elf18 treatment in *N*. *benthamiana* leaves co-expressing PP2C38-FLAG and EFR-GFP (Fig 5A). Intriguingly, we also noted that PP2C38-FLAG shows a constitutive band shift when BIK1 was over-expressed (Fig 1D and S4 Fig). Please note that a band shift is not observed in Fig 1C due to the high molecular weight of the GFP tag.

Protein phosphorylation can often alter migration on SDS-PAGE and can result in band shifts. To test if PP2C38 band shift is due to phosphorylation, we incubated immunoprecipitated PP2C38-FLAG protein from elf18-treated *N*. *benthamiana* leaves with calf intestine alkaline phosphatase (CIP). This dissipated elf18-induced PP2C38-FLAG band shift (Fig 5B), indicating that the higher molecular weight band corresponds to a phosphorylated form of PP2C38.

To identify PP2C38 phosphorylated residues *in vivo*, we analysed immunoprecipitated PP2C38-FLAG protein phosphorylation using liquid chromatography-tandem mass spectrometry (LC-MS/MS) and identified the serine residue 77 (S77) as being phosphorylated (Fig 5C). Interestingly, GST-BIK1 could trans-phosphorylate MBP-PP2C38, and this phosphorylation was mostly S77-dependent, as it was strongly reduced when this serine was substituted to the non-phosphorylatable residue alanine (S77A) (Fig 5D). Consistent with its ability to trans-phosphorylate PP2C38, GST-BIK1 directly interacted with MBP-PP2C38 in an *in vitro* pull down assay (S9 Fig). Furthermore, expression of the PP2C38^S77A^-FLAG variant abolished the band shift of PP2C38-FLAG normally observed upon PAMP treatment in *N*. *benthamiana* (Fig 5E). Similarly, PP2C38^S77A^-FLAG did not show a band shift in response to elf18 in *Arabidopsis* Col-0 protoplasts (Fig 5F). Interestingly, the S77A mutation partially compromised PP2C38 catalytic activity, suggesting that phosphorylation may be important to modulate its function (S10 Fig). Together, our *in vitro* and *in vivo* data suggest that S77 is a major PP2C38 residue phosphorylated by BIK1 after PAMP perception.

**Fig 6:**
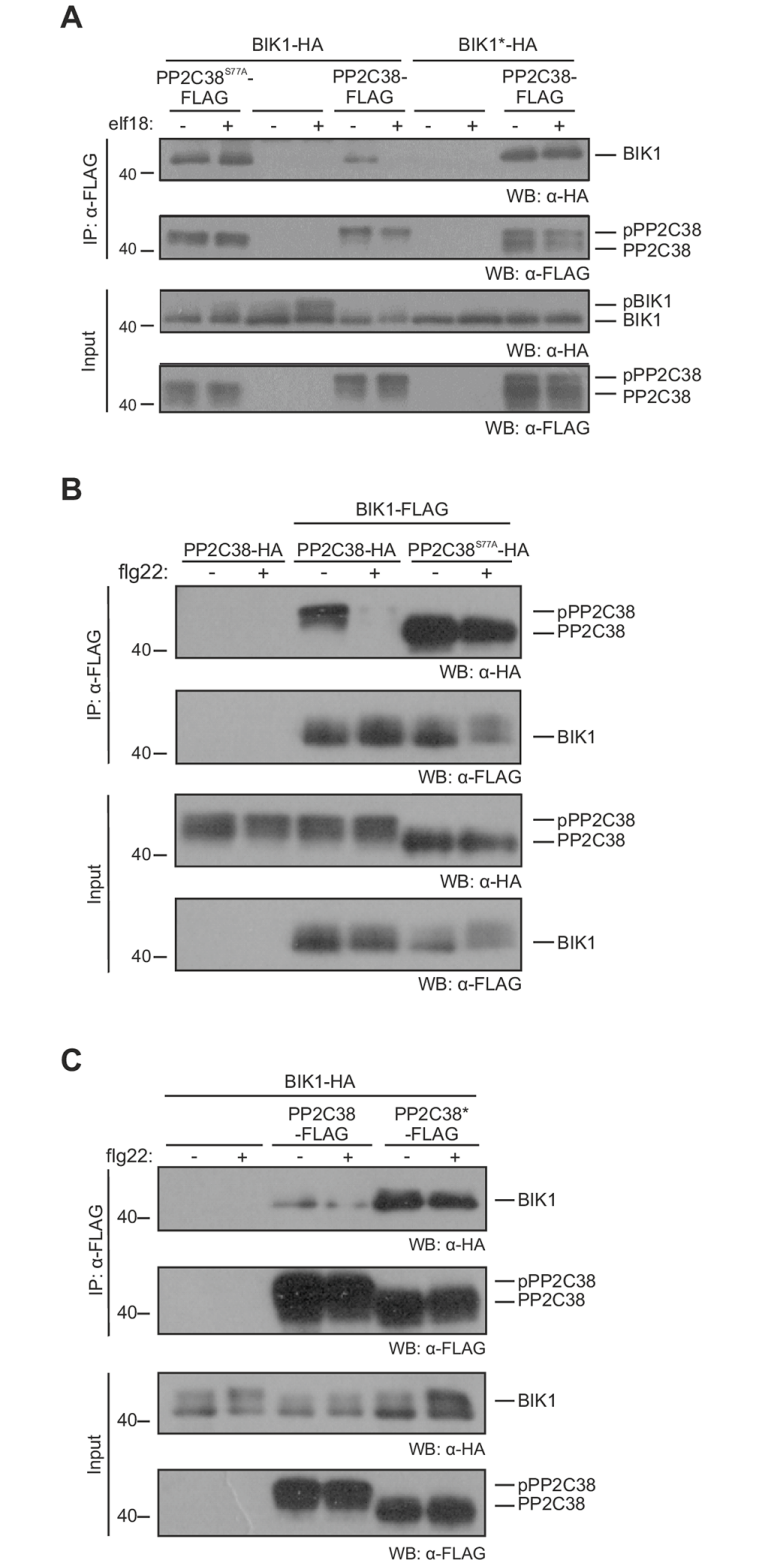
Phosphorylation and catalytic activity are required for PP2C38-BIK1 dissociation. (A-C) Dissociation of PP2C38-BIK1 complex after PAMP perception. *Arabidopsis* Col-0 protoplasts were co-transfected with PP2C38-FLAG/HA or indicated variants together with BIK1/BIK1*-HA/FLAG, and treated (+) or not (-) with 1 μM elf18/flg22 for 30 min. PP2C38-BIK1 complexes were analysed by immunoblotting following α-FLAG immunoprecipitation. Each experiment repeated at least three times with similar results.

We next tested whether S77 phosphorylation may be linked to the dissociation of PP2C38 from the BIK1 complex. We co-expressed PP2C38-FLAG or PP2C38^S77A^-FLAG with BIK1-HA or BIK1*-HA [BIK1* being the kinase-dead variant BIK1^K105E^[24]] in *Arabidopsis* protoplasts. After α-FLAG immunoprecipitation, we detected a clear association of PP2C38 with BIK1 that was disrupted after elf18 treatment (Fig 6A), as previously observed (Fig 1E). In contrast, the PP2C38^S77A^ variant remained stably associated with BIK1, even after elf18 treatment (Fig 6A). Importantly, expression of BIK1* reduced the overall PP2C38 phosphorylation (observed as an increase of the non-phosphorylated form—lower molecular weight), and prevented its dissociation (Fig 6A). These results point towards an important role of PP2C38 phosphorylation and BIK1 kinase activity for the dissociation of the PP2C38-BIK1 complex.

While performing reciprocal co-immunoprecipitation experiments, we detected both the phosphorylated and non-phosphorylated forms of PP2C38 in BIK1 immunoprecipitates, which then dissociated following flg22 treatment (Fig 6B). In turn, BIK1 stably associated with PP2C38^S77A^ even after flg22 treatment (Fig 6B). In addition, we found that PP2C38* did not dissociate from BIK1 following flg22 treatment (Fig 6C). This indicated that while phosphorylation plays an important role in this process, it is not sufficient by itself to drive PP2C38 and BIK1 apart, and suggest a potential link between PP2C38 phosphatase activity and phosphorylation by BIK1.

## Discussion

Appropriate initiation, timing and amplitude of immune signalling must be carefully regulated to avoid excessive or nonspecific activation of immune responses, which can lead to autoimmune and inflammatory diseases [49, 50]. The mechanisms and pathways that negatively regulate PRR-triggered immunity (PTI) in mammals have been extensively characterized [49, 51, 52]. However, much less is known in plants, where a fine balance between immunity and growth is important for their optimal reproductive success [53, 54].

BIK1 is a central positive regulator of immune signalling acting downstream of both LRR- and LysM-containing PRRs [19–21]. Yet, despite the importance of BIK1 for plant immunity, knowledge concerning its action, substrates or regulation is still sparse. The first example of a downstream BIK1 substrate was the NADPH oxidase RBOHD, which generates PAMP-induced ROS burst [23, 24]. Notably, PAMP-activated BIK1 directly phosphorylates RBOHD, which is required for ROS production and subsequent stomatal immunity [23, 24]. Importantly, BIK1 turnover was recently shown to be controlled by the calcium-dependent protein kinase CPK28 and heterotrimeric G proteins in a proteasome-dependent manner [36, 37], which provides one mechanism by which plant cells control this key regulator and the amplitude of immune signalling. Yet, the regulation imposed by CPK28 appears to be constitutive, as no effect of PAMP treatment could be noted on CPK28 activity or association with BIK1 [36], and thus seems to represent a mechanism used by plant cells to constantly buffer plant immune signalling. In this work, we revealed the protein phosphatase type 2C PP2C38 as a dynamic regulator of BIK1, which controls its phosphorylation status, most likely to maintain basal immune signalling levels at a minimum in the absence of elicitation and/or to fine-tune the immune responses upon pathogen attack.

PP2C38 was initially identified in an Y2H screen as an interactor of EFR (S1 Table). While we confirmed that PP2C38 associates with EFR (as well as with FLS2), we also found that PP2C38 associates with BIK1 *in planta* (Fig 1) and that both proteins directly interact *in vitro* (S9 Fig). Notably, no association could be observed between PP2C38 and BAK1 (Fig 1 and S3 Fig). PP2C38 is an active PM-localized phosphatase (Fig 2). Interestingly, *PP2C38* over-expression affected elf18-induced BIK1 hyper-phosphorylation, but had no impact on EFR or BAK1 phosphorylation (Fig 3). These results suggest that PP2C38 directly dephosphorylates BIK1, and that BIK1 is a biologically relevant substrate of PP2C38. This is further substantiated by our findings that *PP2C38* over-expression leads to a reduction of PAMP-induced phosphorylation of RBOHD on the BIK1-specific phosphosite S39 (Fig 3C). Consequently, ectopic *PP2C38* expression led to a significant reduction of PAMP-triggered ROS burst in *N*. *benthamiana* and *Arabidopsis* plants (Fig 4A and 4B) and compromised PAMP-induced stomatal closure resulting in enhanced susceptibility to a non-pathogenic *P*. *syringae* strain (Fig 4D and 4E). In addition, loss of PP2C38 and its paralog PP2C48 led to enhanced ROS productions in response to elf18 and flg22, as well as to increased resistance against *P*. *syringae hrcC* ^*-*^ strain (Fig 4C and 4F). Interestingly, *pp2c48-1* single mutants also exhibited higher ROS in response to flg22 (S7 Fig), suggesting that PP2C38 and PP2C48 may play differential roles according to the eliciting stimulus. Together, these results implicate PP2C38 (and PP2C48) as negative regulator(s) of BIK1-mediated immune signalling and stomatal anti-bacterial immunity.

We observed that PP2C38 becomes phosphorylated and dissociates from the PRR complex upon PAMP treatment (Figs 1 and 5). PP2C38 phosphorylation is important for its dissociation from BIK1, as the non-phosphorylatable PP2C38^S77A^ variant could not dissociate from BIK1 after elf18 perception (Fig 6A). Over-expression of BIK1 resulted in a constitutive PP2C38 band shift in *N*. *benthamiana* leaves and in *Arabidopsis* protoplasts (Figs 1, 5 and S3 Fig). Moreover, PP2C38 failed to dissociate from a kinase-dead BIK1 variant (carrying the K105E mutation), which is known to be dominant-negative [24], and exhibited reduced phosphorylation levels (Fig 6A). In addition, EFR or FLS2 kinase activity is not required for PP2C38 dissociation (S11 Fig), and BAK1 does not associate with PP2C38 *in planta* (Fig 1 and S3 Fig). Furthermore, BIK1 can trans-phosphorylate PP2C38 *in vitro* in a manner that mostly depends on S77 (Fig 5C). Altogether, this suggests that BIK1 is responsible for PP2C38 phosphorylation.

Phosphorylated PP2C38 could still associate with BIK1 in the absence of PAMP treatment (Fig 6B), suggesting that phosphorylation is not sufficient for PP2C38 dissociation and that additional mechanisms must play a role in this process. Moreover, the catalytic-dead PP2C38* variant could not dissociate from BIK1 after PAMP treatment (Fig 6C). Intriguingly, PP2C38* migrated as a lower molecular weight band in SDS-PAGE, consistent with the size of non-phosphorylated PP2C38 (Figs 3 and 6). While *in vitro* experiments revealed that PP2C38* could be phosphorylated by BIK1 (Fig 5E); it is difficult to conclude whether PP2C38* is phosphorylated *in vivo*, or if mutating the two catalytic aspartic acid residues (negatively charged) into asparagines (positively charged) leads to an overall change in the isoelectric point averting the mobility shift. It is thus possible that, in addition to S77 phosphorylation, the catalytic activity of PP2C38 may be required for its dissociation from BIK1 following PAMP perception.

**Fig 7:**
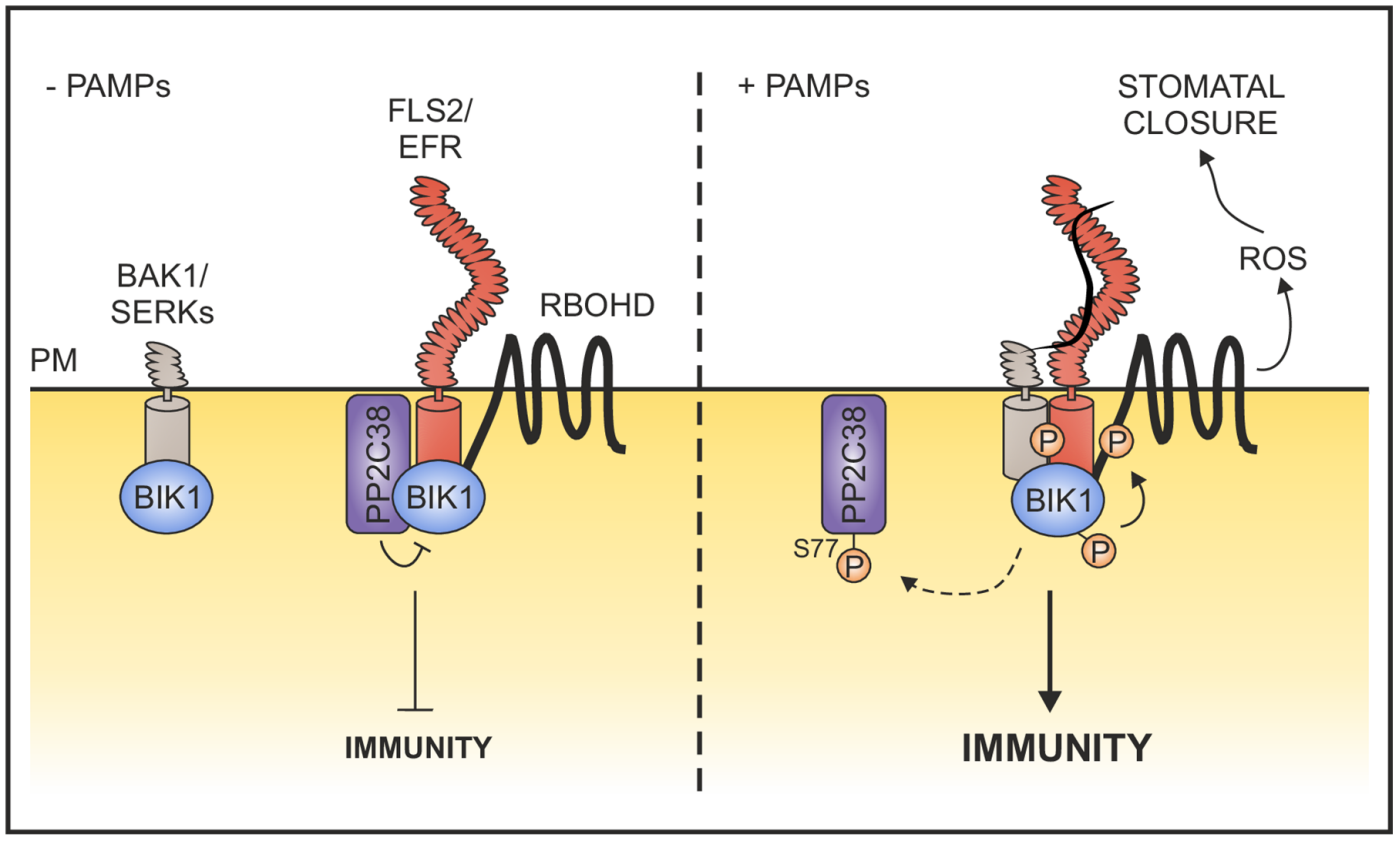
Model: PP2C38 dephosphorylates BIK1 to attenuate innate immune signalling. In the absence of pathogen elicitation, PP2C38 associates with PRR-BIK1 complexes to dephosphorylate BIK1 and prevent its activation. Upon PAMP perception, BAK1 is recruited and trans-phosphorylation of the PRR complex triggers BIK1 hyper-phosphorylation. In turn, activated BIK1 presumably phosphorylates PP2C38 on S77 to enable its dissociation from the PRR complex. The release of PP2C38 relieves the negative regulation imposed on BIK1, allowing efficient subsequent activation of downstream targets.

We therefore propose a model in which PP2C38 associates with BIK1 in the resting state, keeping its phosphorylation status under control. Upon PAMP perception, BAK1 forms a stable complex with EFR/FLS2, resulting in trans-phosphorylation between these proteins and BIK1, leading to BIK1 activation. Consequently, PP2C38 is phosphorylated and released from BIK1 allowing its full activation (Fig 7). The catalytic activity of PP2C38 is likely to play an important role in this process, however additional work will need to be performed in order to fully understand this molecular mechanism. The model proposed is somewhat reminiscent of the negative regulation imposed by the PM-anchored protein BKI1 on the brassinosteroid (BR) receptor BRI1. BKI1 interacts with BRI1 kinase domain preventing its phosphorylation [55–57]. BR perception by BRI1 results in BKI1 phosphorylation, which triggers BKI1 dissociation and relocalization to the cytoplasm [53]. However, in the case of PP2C38 such ligand-dependent relocalization does not seem to occur (Fig 2D), suggesting that PP2C38 is associated to the PM, possibly due to post-translation modifications such as palmitoylation. Notably, BIK1 also integrates signalling from BRI1, acting as a negative regulator of BR-dependent responses [58]. Whether PP2C38 also negatively regulates BIK1 to control BR responses (in this case, to potentially activate them) remains to be determined. In the process of our work however, we have not observed a growth phenotype suggesting a potential role of PP2C38 in BR signalling.

Despite the important role of protein phosphorylation for PRR complexes, only a small number of protein phosphatases have been shown previously to act at the PRR level. The unclustered PP2C KAPP interacts with FLS2 cytoplasmic domain in yeast two-hybrid assays and *KAPP* over-expression results in flg22 insensitivity [59]. However, KAPP is known to interact with several unrelated RKs [60], which questions the specificity of this action. In rice, the PP2C XB15 associates with the PRR XA21 and negatively regulates XA21-mediated resistance to *Xoo* [61]. Interestingly, the *Arabidopsis* XB15 orthologues PLL4 and PLL5 associate with EFR (which is phylogenetically closely related to XA21) and negatively regulate EFR-mediated responses, demonstrating the conservation of regulatory mechanisms between evolutionary-distant plants [62]. Recently, a specific protein phosphatase 2A (PP2A) holoenzyme (composed of the subunits A1, C4 and B’η/ζ) was shown to negatively regulate PTI by directly targeting the co-receptor BAK1 [16]. While BAK1-associated PP2A activity was reduced after PAMP treatment, it is still unclear whether this is due to the dissociation of PP2A from BAK1, or to the inhibition of PP2A activity. The identification of PP2C38 as a regulator of BIK1 further illustrates the negative regulation of plant immune signalling that occurs at multiple levels within PRR complexes.

## Materials and Methods

### Yeast two-hybrid screen

The EFR cytoplasmic domain (EFR-CD) was amplified from cDNA, cloned into the pAs-attR yeast two-hybrid screening bait plasmid and transformed into yeast strain AH109 (MATa). A cDNA library was generated from two-weeks-old *Arabidopsis* seedlings treated for 30 min with 10 μM flg22, and cloned into pACT-attR yeast two-hybrid screening prey plasmids. Together with a cDNA library derived from *Arabidopsis* cell suspension culture [63], as well as a commercially available cDNA library derived from *Arabidopsis* plants (Clontech), these three libraries were screened against EFR-CD by interaction mating as described previously [64]. A total of eight million zygotes were screened, and 47 candidate interaction partners were obtained, facilitating yeast growth on triple-dropout media (lacking leucine, tryptophan and histidine, supplemented with 5 mM 3-Amino triazole). Three clones were identified as PP2C38, and five clones matched PP2C58.

### Plant material and growth conditions

*Arabidopsis* plants used for ROS burst, stomatal closure and infection assays were grown in soil at 21°C with a 10-h photoperiod. For *Arabidopsis* sterile seedlings, seeds were surface-sterilized with chlorine gas and germinated on plates containing Murashige-Skoog (MS) medium (including vitamins; Duchefa) and 1% sucrose supplemented with 0.8% agar for the first 5–7 days at 22°C and with a 16-h photoperiod. Seedlings were transferred to liquid MS medium supplemented with 1% sucrose and grown under the same conditions for 7–10 days.

*Nicotiana benthamiana* plants were soil-grown under a 16 h photoperiod at 22°C. *Arabidopsis* lines used: Col-0 (WT), *fls2* [6], *efr* [65], *cerk1* [66] *efr*/*pEFR*:*EFR-GFP* [67], *pp2c38-1* (SALK_036920), and *pp2c48-1* (SALKseq_061058) (this study).

### Chemicals and elicitors

Flg22 and elf18 peptides were purchased from EZBiolab. ABA was purchased from Sigma-Aldrich. Phosphatase inhibitors NaF and Na2MoO4 purchased from Sigma-Aldrich. For clarity, we used flg22 and elf18 for most phenotypic experiments in Arabidopsis, flg22 for most phenotypic and biochemical experiments in *N*. *benthamiana* (as EFR is not present in this plant species), and elf18 for most experiments in Arabidopsis protoplasts.

### Molecular cloning and generation of *PP2C38* over-expression lines

For the site-directed mutagenesis of *PP2C38*, DNA fragments were amplified by PCR using primers harbouring desired mutation from pENTR-PP2C38 (see S2 Table for full list of primers used in this study). For recombinant protein purification, *PP2C38* CDS was inserted between BamHI and SalI restriction sites of pMAL-c4E (NEB). For the generation of *PP2C38* over-expression lines, *PP2C38* CDS was fused to GFP in the gateway-compatible pK7GW2.0 vector (Invitrogen). The *Agrobacterium tumefaciens* strain GV3101 was used to generate stable *Arabidopsis* lines. Homozygous transgenic plants were obtained base on segregation ratios on selection plates.

### RNA isolation and qRT-PCR

Gene expression was analysed on 2-week-old *Arabidopsis* seedlings grown in liquid MS medium. Total RNA was extracted as described in [68]. First-strand cDNA synthesis was performed with SuperScript III RNA transcriptase (Invitrogen) and oligo(dT) primer, according to the manufacturer’s instructions. The qPCR was performed using SYBR Green JumpStart Taq ReadyMix (Sigma-Aldrich) in a BioRad CFX96 real-time system. The relative expression values were determined by using U-box gene (*At5g15400*) as reference and the comparative Ct method (2-ΔΔCt). Primers used for quantitative PCR are listed in S1 Table.

### Protein extraction and immunoprecipitation

*N*. *benthamiana* leaves or *Arabidopsis* seedlings were ground to a fine powder in liquid nitrogen with sand (Sigma-Aldrich). Proteins were extracted in buffer containing 50 mM Tris-HCl, pH 7.5, 150 mM NaCl, 10% glycerol, 10 mM DTT, 1 mM NaF, 1 mM Na2MoO4.2H2O, 1% Phosphatase Inhibitor Cocktails 2 and 3 (Sigma-Aldrich), 1% (v/v) P9599 Protease Inhibitor Cocktail (Sigma-Aldrich), 100 μM phenylmethylsulphonyl fluoride and 0.5% (v/v) IGEPAL CA-630 (Sigma-Aldrich). Extracts were incubated 30 min at 4°C and centrifuged for 20 min at 16,000 *g* at 4°C. Supernatants were incubated for 1–2 h at 4°C with GFP-Trap (ChromoTek) or ANTI-FLAG M2 Affinity Gel (Sigma-Aldrich), and washed 3–4 times with extraction buffer containing 0.1–0.5% (v/v) IGEPAL CA-630 (Sigma-Aldrich). For GFP-Trap immunoprecipitation, beads were boiled in NuPAGE LDS sample buffer (Thermo Scientific) to release proteins. FLAG peptide was used for specific elution of anti-FLAG immunoprecipitated proteins. For immunoprecipitation in *Arabidopsis* protoplasts, protoplasts were transfected with indicated plasmids, incubated overnight and then treated with H_2_O or 1 μM flg22 or elf18 for 15–30 min. Proteins were extracted with extraction buffer (50 mM HEPES [pH 7.5], 150 mM KCl, 1 mM EDTA, 0.5% Trition-X 100, 1 mM DTT, proteinase inhibitor cocktail) at 4°C. Immunoprecipitation was then carried out as described above.

### *In vitro* pull-down

Recombinant proteins were incubated in column buffer (50 mM Tris [pH 7.5], 50 mM NaCl, 0.2% IGEPAL CA-630) for 15 min at 4°C and centrifuged for 2 min at 16,000 g. The supernatant was recovered and the proteins incubated for 30 min at 4°C with amylose resin (NEB) for MBP pull-down. Resin was washed three times with column buffer and boiled in SDS sample buffer before loading in SDS-PAGE.

### Mass spectrometry

Proteins were separated by SDS-PAGE (NuPAGE, Invitrogen), and after staining with Coomassie brilliant Blue G-250 CBB (SimplyBlue stain, Invitrogen) the proteins were cut out and digested by Trypsin and AspN. LC-MS/MS analysis was performed using an LTQ-Orbitrap mass-spectrometer (Thermo Scientific) and a nanoflow-HPLC system (nanoAcquity; Waters) as described previously [23]. The Arabidopsis database (TAIR10) was searched using Mascot (v 2.4.1 Matrix Science). Parameters were set for 10 ppm peptide mass tolerance and allowing for Met oxidation and three missed cleavages. Carbamidomethylation of Cys residues was specified as a fixed modification, and oxidized Met and phosphorylation of Ser, Tyr or Thr residues were allowed as variable modifications. Scaffold (v4; Proteome Software) was used to validate MS/MS-based peptide and protein identifications and annotate spectra. The position and quality of spectra for phosphopeptides were also manually examined before acceptance. In the 3 independent PP2C38-FLAG IP samples we analysed, we measured a range of 71–85% coverage from around 75–100 unique peptides (250–400 spectral counts total). We predominately measured phosphorylation of S77 with on average 10–15 spectral counts in each sample.

### Immunoblotting

Protein samples were typically separated in 8–10% bisacrylamide gels at 120–140 V. For analysis of PP2C38 band shift, 12% gels were run at 100V for 3:30-4h. Immunoblotting was performed with antibodies diluted in blocking solution (5% fat-free milk in TBS with 0.1% [v/v] Tween-20). Antibodies used in this study: α-BAK1 [13]; α-FLS2 [8]; α-pS39-RBOHD [24]; α-HA-horseradish peroxidase (HRP) (3F10, Roche); α-GFP-HRP (B-2, Santa Cruz); α-FLAG-HRP (M2 monoclonal antibody, Sigma-Aldrich). Blots were developed with Pierce ECL/ ECL Femto Western Blotting Substrate (Thermo Scientific).

### Kinase and phosphorylation assays

Immunoprecipitated or recombinant proteins were incubated in kinase buffer (50 mM Tris-HCl, pH 7.5, 5 mM MnCl_2_, 1 mM DTT) and supplemented with 1 μM unlabeled ATP and 183 kBq of [^32^P]γ-ATP for 30–60 min at 30°C with shaking. For trans-phosphorylation assays, the phosphorylated kinase was then incubated with substrate protein for 30 min. Reactions were stopped by adding SDS sample buffer and heating at 70°C for 15 min. Proteins were separated by SDS-PAGE and transferred onto PVDF membranes (Biorad), followed by staining with CBB. Phosphorylation was analysed by autoradiography using a FUJI Film FLA5000 PhosphoImager (Fuji, Tokyo, Japan).

### *In vitro* phosphatase assay

Phosphatase assays were performed using the Serine/Threonine Phosphatase Assay System (Promega) according to the manufacturer’s instructions.

### ROS burst assay

Leaf discs (4 mm diameter) from 4- to 5-week-old *Arabidopsis* or *N*. *benthamiana* plants were collected and ROS measurements performed as previously described [23]. PAMP treatments, as described in figure legends, were applied immediately before luminescence was captured over 40–60 min using either a Varioskan Flash (Thermo Scientific) multiplate reader or a Photek camera (East Sussex, UK).

### Stomatal aperture measurements

Stomatal aperture measurements were performed as described [48].

### Bacterial infection assays

Five-week old plants were sprayed with a solution of *Pseudomonas syringae* pv. *tomato* (*Pto*) DC3000 *hrcC* ^*-*^ (0.5×10^9^ CFU/ml) supplemented with 0.04% Silwett L-077, and bacterial numbers determined 4 days post infection by serial dilution plating.

### Confocal microscopy

Confocal micrographs were acquired on a Leica TCS SP5 confocal microscope. GFP was excited at 488 nm using an argon laser. Fluorescence emission was collected between 500–540 nm. Image analysis was carried out with FIJI (ImageJ).

### Statistical analyses

Linear Mixed Effect Modelling of ROS data was carried out in the R programming language [69] version 3.2.0 in R Studio 0.98 using the nlme package [70] as shown in the code described in S1 Appendix (ros_analysis_elf18.Rmd and ros_analysis_flg22.Rmd).

## Supporting Information

S1 Fig PP2C58 does not associate with EFR *in planta*. (A-B) Co-immunoprecipitation of FLAG-tagged (A) or HA-tagged (B) PP2C58 and EFR proteins transiently expressed in *N*. *benthamiana* leaves treated (+) or not (-) with 100 nM elf18.
(TIF)

S2 Fig Characterization of PP2C38 over-expression and mutant lines. (A) Gene structure of *PP2C38* and *PP2C48* showing position of exons (boxes), introns (lines) and T-DNA insertion sites (triangle); arrows indicate position of primers used for genotyping. (B) *PP2C38* expression analysis by quantitative RT-PCR. Expression was normalized to *UBQ10* and Col-0. (C) *PP2C48* expression analysis by quantitative RT-PCR. Expression was normalized to *UBQ10* and Col-0.
(TIF)

S3 Fig *BIK1* over-expression results in constitutive PP2C38 band shift. PPC2C38-FLAG and BIK1-HA proteins were co-expressed in *N*. *benthamiana* leaves and treated (+) or not (-) with 100 nM flg22. Upper band corresponds to phosphorylated PP2C38 form (pPP2C38). 12% bisacrylamide gels were used for better protein separation. Experiment repeated three times with similar results.
(TIF)

S4 Fig PP2C38 does not associate with BAK1 *in planta*. PP2C38-FLAG and BIK1-HA proteins were transiently expressed in *Arabidopsis* Col-0 protoplasts treated (+) or not (-) with 1 μM elf18. Endogenous BAK1 was detected using α-BAK1 antibody. Co-immunoprecipitation reveals that PP2C38 dynamically associates with BIK1 but not with BAK1.
(TIF)

S5 Fig Phylogenetic analysis of PP2C38 orthologs. Distance trees are based on protein sequences aligned with MUSCLE (produced with SEAVIEW, using neighbor joining). Protein sequences retrieved from pBLAST search using PP2C38 as query. Nomenclature of *Arabidopsis* and *O*. *sativa japonica* proteins according to Xue et al. (2008) [38]; nomenclature of proteins from other plant species according to UniProt identifiers.
(TIF)

S6 Fig PAMP-induced ROS burst on *PP2C38* over-expression lines. Two independent Arabidopsis transgenic lines expressing 35S::*PP2C38-GFP* in the *pp2c38-1* show reduced ROS burst induced by 10 nM flg22 (upper panel) and elf18 (lower panel). Values are mean ± SE (n = 12) and are expressed in relative light units (RLU). Asterisks indicate significant differences compared to Col-0 (one-way ANOVA, Dunnet post hoc test, ***p < 0.001; **p < 0.01; *p < 0.05). Experiment replicated three times with similar results.
(TIF)

S7 Fig PAMP-induced ROS burst on *PP2C38* and *PP2C48* mutant lines. (A-B) ROS production in *pp2c38-1* and *pp2c48-1* single and double mutants in response to 100 nM elf18 (A) or flg22 (B). Scatter and notched boxplots display values of total photon counts, scaled as a proportion of the intensity in the corresponding Col-0 experiment performed at the same time, with different colours representing 4–5 independent experiments. Statistical analysis performed using linear mixed effects model implemented in the R statistical programming language. Letters indicate significantly different means at p < 0.05 after Holm’s correction.
(TIF)

S8 Fig PP2C38 regulates flg22-induced stomatal closure. Stomatal aperture was measured 2 h after 5 μM flg22. Values are mean ± SE (n>60; one-way ANOVA, Tukey post hoc test). Different letters indicate significantly different values at *p* < 0.001. Experiment replicated three times with similar results.
(TIF)

S9 Fig PP2C38 directly interacts with BIK1 *in vitro*. *In vitro* MBP pull-down performed with recombinant MBP-PP2C38 and GST-BIK1. Free MBP and GST proteins used as control. Experiment repeated three times with similar results.
(TIF)

S10 Fig Catalytic activity of PP2C^S77A^ variant. Recombinant MBP-PP2C38, MBP-PP2C38^S77A^ or MBP-PP2C38* proteins were incubated with a synthetic phosphopeptide in the presence or absence of Mg^2+^ ions. Release of inorganic phosphate was quantified using a colorimetric assay. Values are averages ± SD (n = 3). Letters indicate statistically significant differences based on ANOVA, Dunnet post hoc test, *p* < 0.001.
(TIF)

S11 Fig EFR and FLS2 kinase activities are not required for PAMP-induced dissociation of PP2C38. (A-B) PP2C38 and kinase-dead versions of EFR* (A) or FLS2* (B) proteins were co-immunoprecipitated from *N*. *benthamiana* leaves treated (+) or not (-) with 100 nM elf18 (A) or flg22 (B) for 20 min.
(TIF)

S1 Table List of proteins interacting with EFR-CD in Y2H screen. (PDF)

S2 Table List of primers used in this study. (PDF)

S1 Appendix Raw data and source code used in the analysis of PAMP-induced ROS production in S7 Fig. (ZIP)
