# Supplementary figures and images for: The Arabidopsis Protein Phosphatase PP2C38 Negatively Regulates the Central Immune Kinase BIK1
Source: PLoS Pathog. 2016 Aug 5;12(8):e1005811. doi: 10.1371/journal.ppat.1005811 (PMC4975489; doi:10.1371/journal.ppat.1005811)

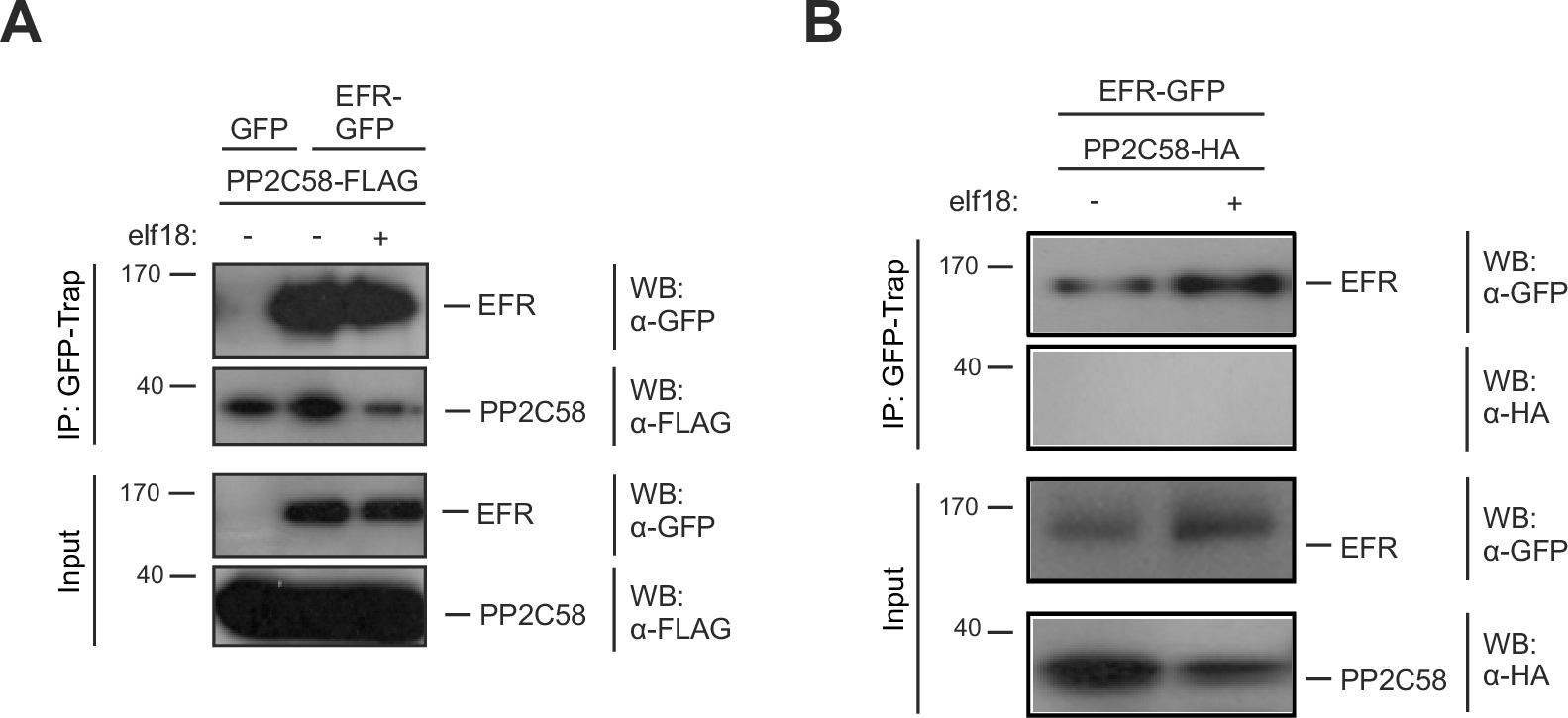

Supplement: S1 Fig — (A-B) Co-immunoprecipitation of FLAG-tagged (A) or HA-tagged (B) PP2C58 and EFR proteins transiently expressed in N. benthamiana leaves treated (+) or not (-) with 100 nM elf18. (TIF) [file ppat.1005811.s001.tif]

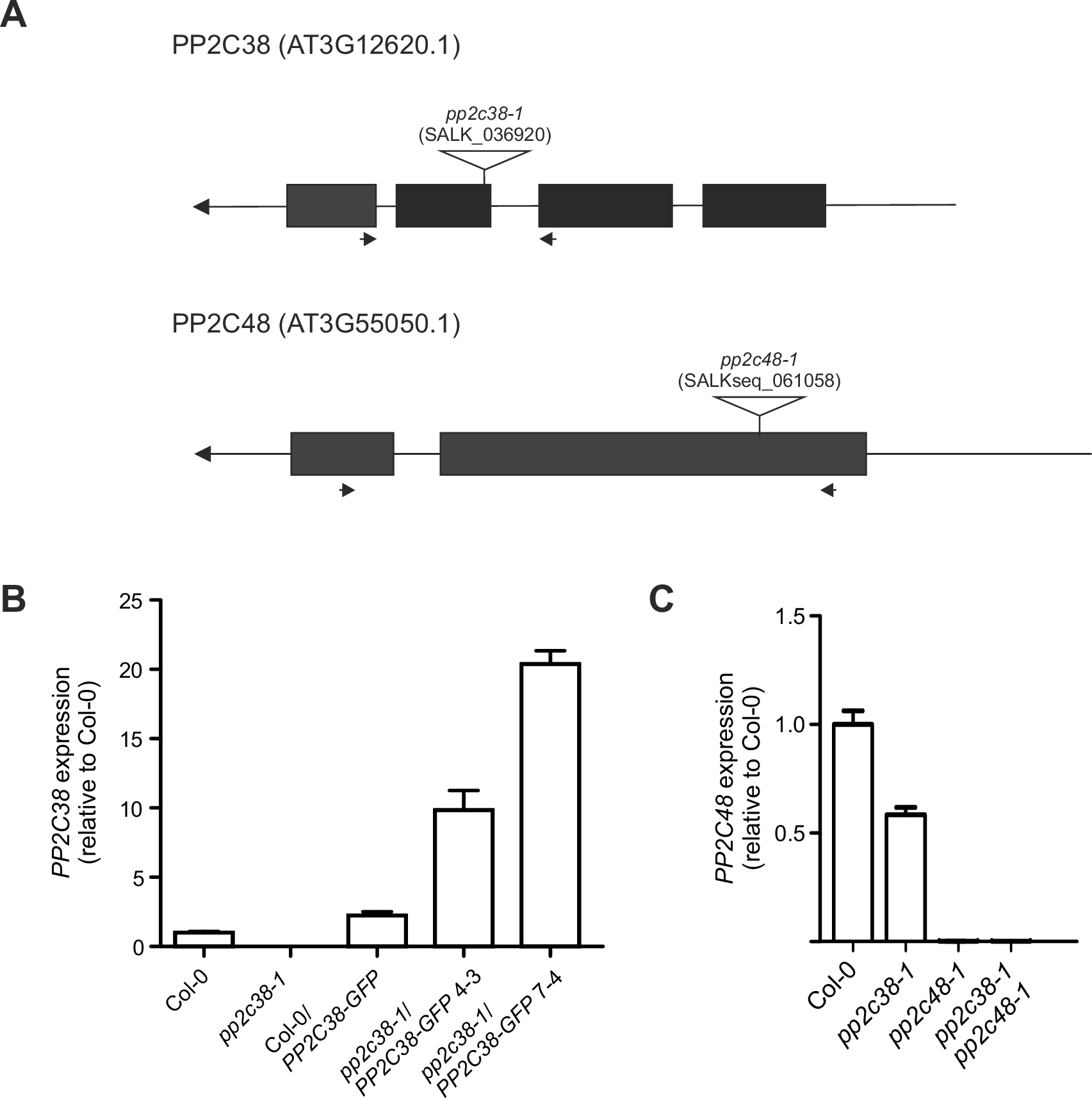

Supplement: S2 Fig — (A) Gene structure of PP2C38 and PP2C48 showing position of exons (boxes), introns (lines) and T-DNA insertion sites (triangle); arrows indicate position of primers used for genotyping. (B) PP2C38 expression analysis by quantitative RT-PCR. Expression was normalized to UBQ10 and Col-0. (C) PP2C48 expression analysis by quantitative RT-PCR. Expression was normalized to UBQ10 and Col-0. (TIF) [file ppat.1005811.s002.tif]

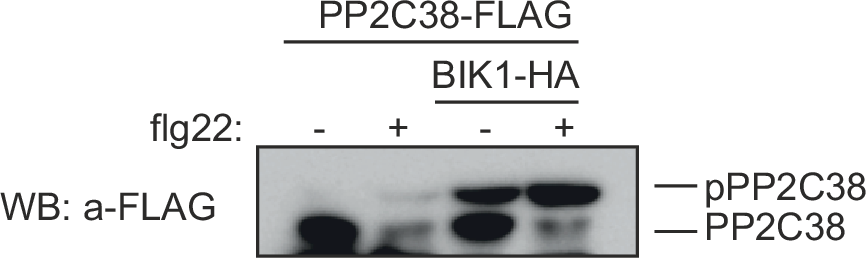

Supplement: S3 Fig — PPC2C38-FLAG and BIK1-HA proteins were co-expressed in N. benthamiana leaves and treated (+) or not (-) with 100 nM flg22. Upper band corresponds to phosphorylated PP2C38 form (pPP2C38). 12% bisacrylamide gels were used for better protein separation. Experiment repeated three times with similar results. (TIF) [file ppat.1005811.s003.tif]

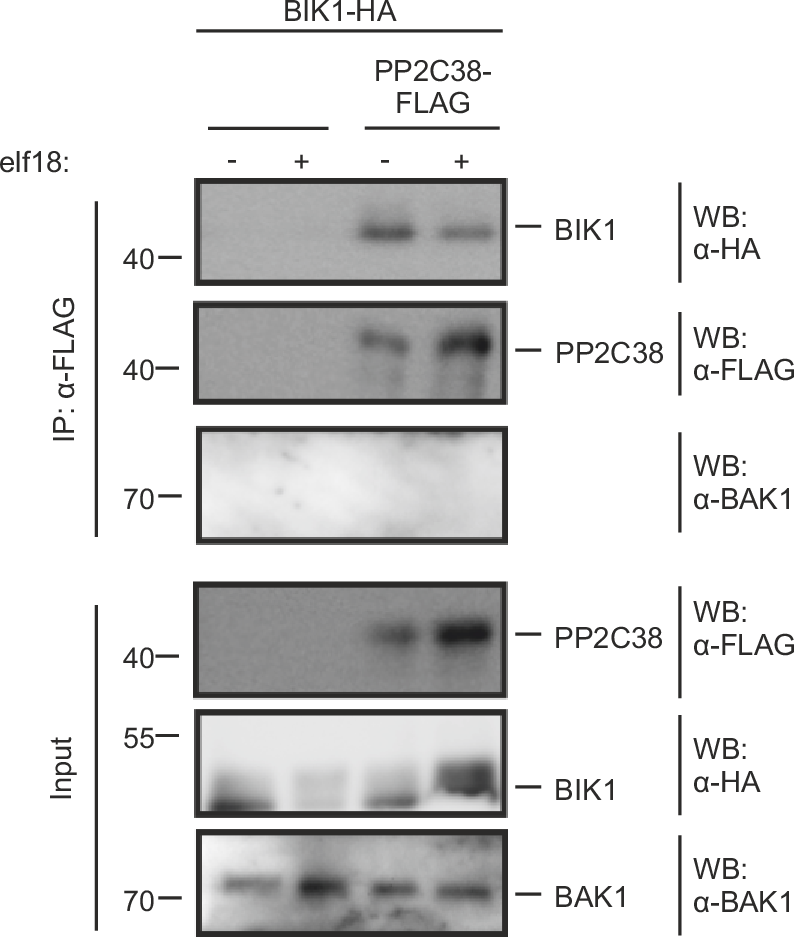

Supplement: S4 Fig — PP2C38-FLAG and BIK1-HA proteins were transiently expressed in Arabidopsis Col-0 protoplasts treated (+) or not (-) with 1 μM elf18. Endogenous BAK1 was detected using α-BAK1 antibody. Co-immunoprecipitation reveals that PP2C38 dynamically associates with BIK1 but not with BAK1. (TIF) [file ppat.1005811.s004.tif]

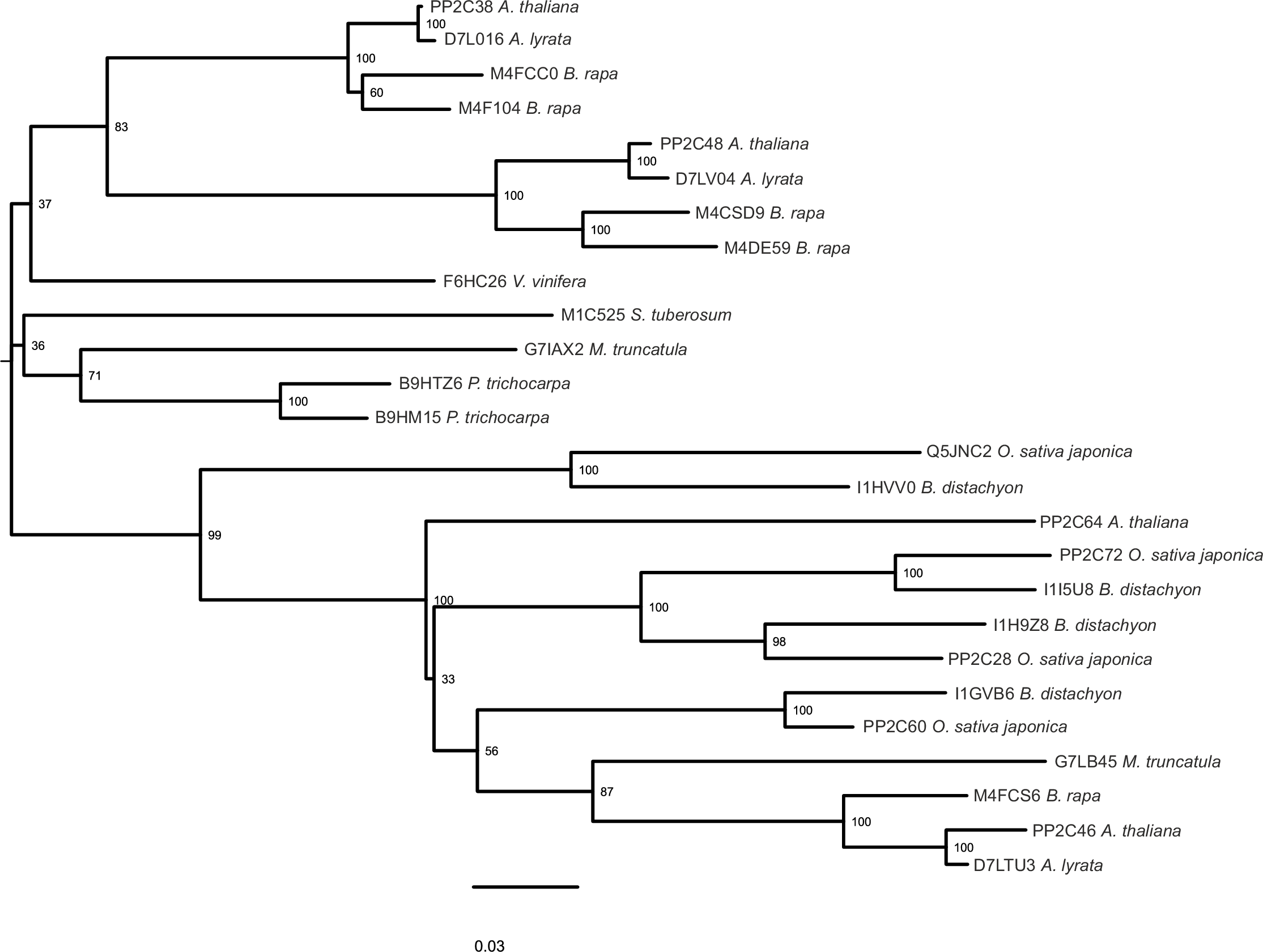

Supplement: S5 Fig — Distance trees are based on protein sequences aligned with MUSCLE (produced with SEAVIEW, using neighbor joining). Protein sequences retrieved from pBLAST search using PP2C38 as query. Nomenclature of Arabidopsis and O. sativa japonica proteins according to Xue et al. (2008) [38]; nomenclature of proteins from other plant species according to UniProt identifiers. (TIF) [file ppat.1005811.s005.tif]

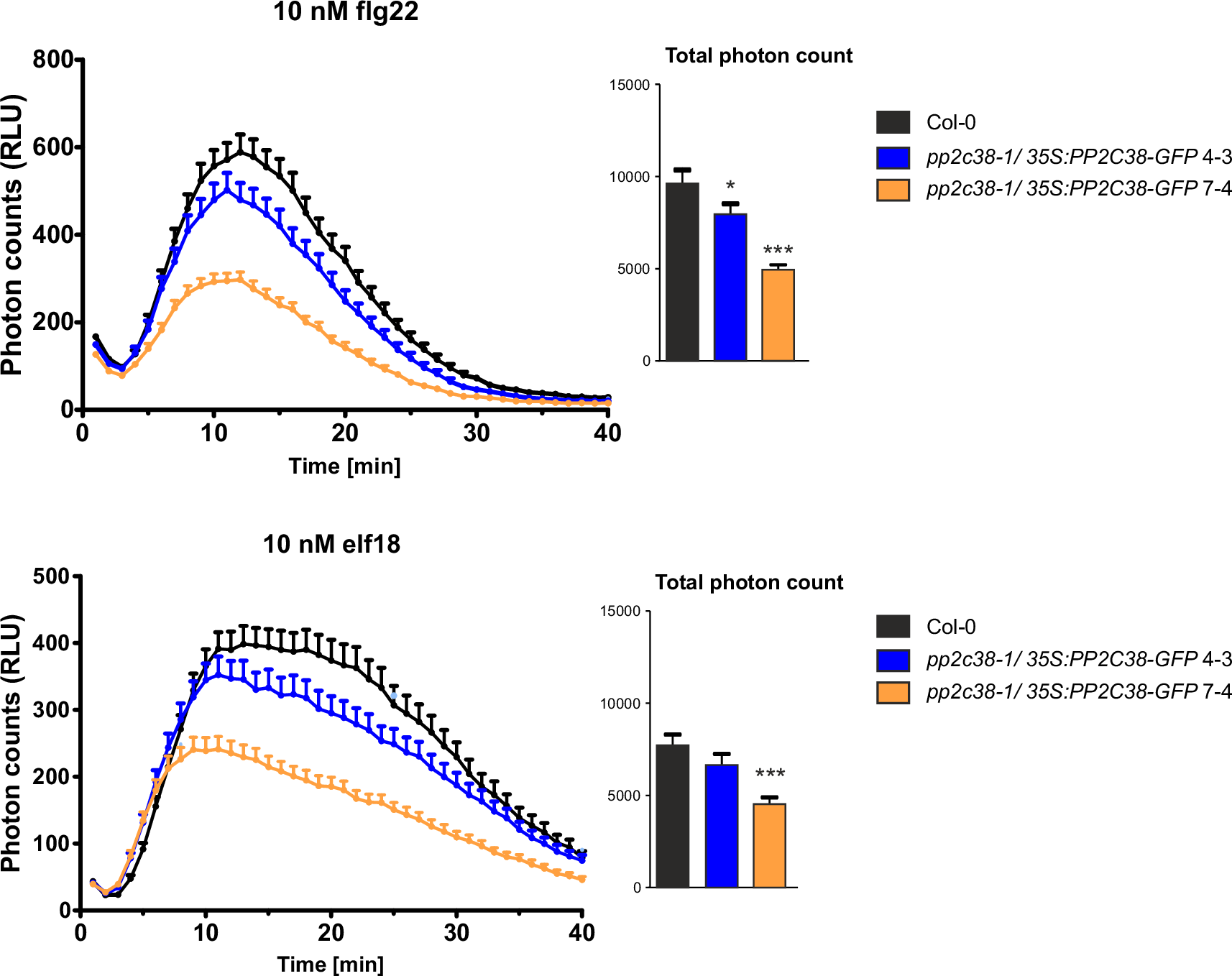

Supplement: S6 Fig — Two independent Arabidopsis transgenic lines expressing 35S::PP2C38-GFP in the pp2c38-1 show reduced ROS burst induced by 10 nM flg22 (upper panel) and elf18 (lower panel). Values are mean ± SE (n = 12) and are expressed in relative light units (RLU). Asterisks indicate significant differences compared to Col-0 (one-way ANOVA, Dunnet post hoc test, ***p < 0.001; **p < 0.01; *p < 0.05). Experiment replicated three times with similar results. (TIF) [file ppat.1005811.s006.tif]

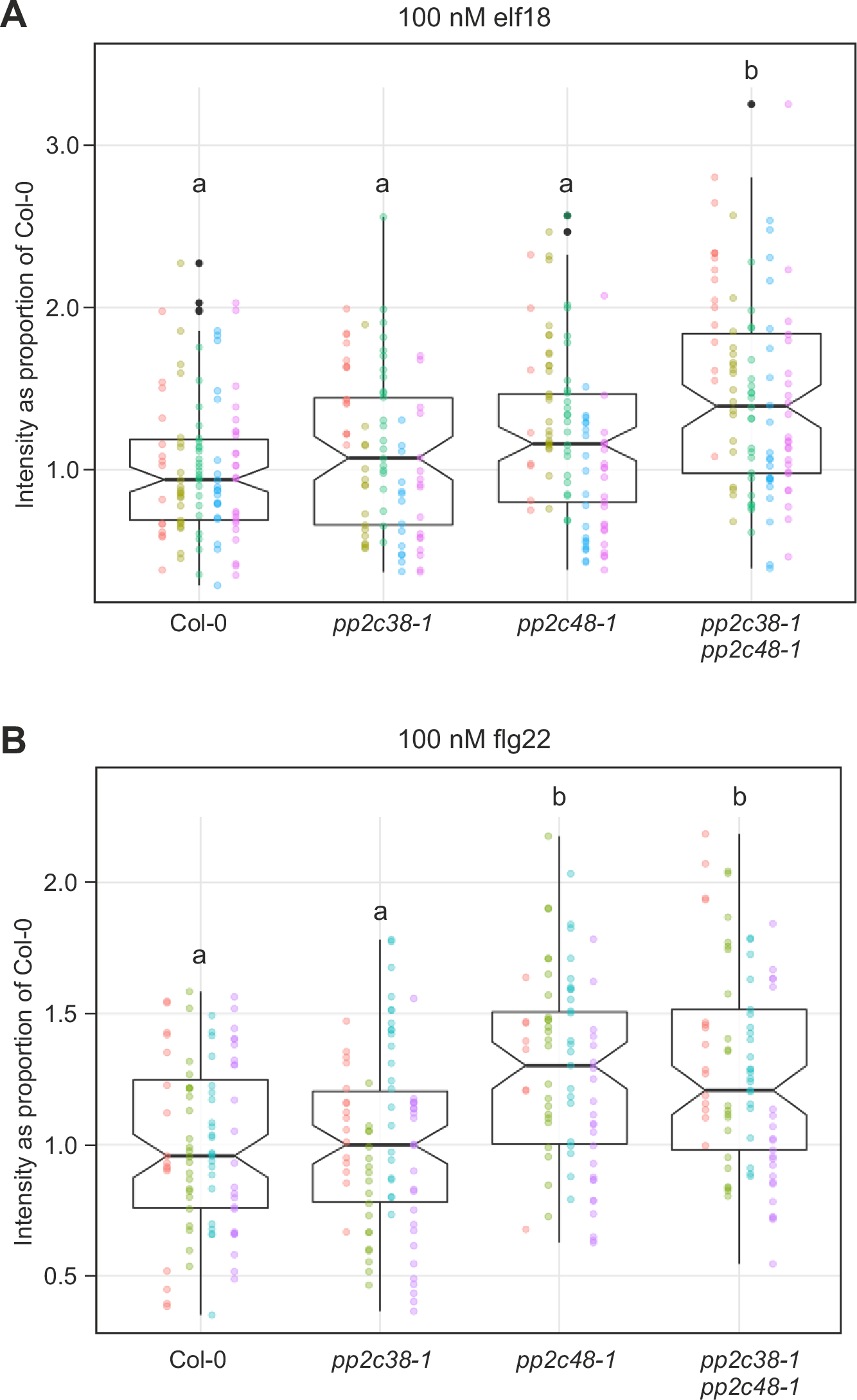

Supplement: S7 Fig — (A-B) ROS production in pp2c38-1 and pp2c48-1 single and double mutants in response to 100 nM elf18 (A) or flg22 (B). Scatter and notched boxplots display values of total photon counts, scaled as a proportion of the intensity in the corresponding Col-0 experiment performed at the same time, with different colours representing 4–5 independent experiments. Statistical analysis performed using linear mixed effects model implemented in the R statistical programming language. Letters indicate significantly different means at p < 0.05 after Holm’s correction. (TIF) [file ppat.1005811.s007.tif]

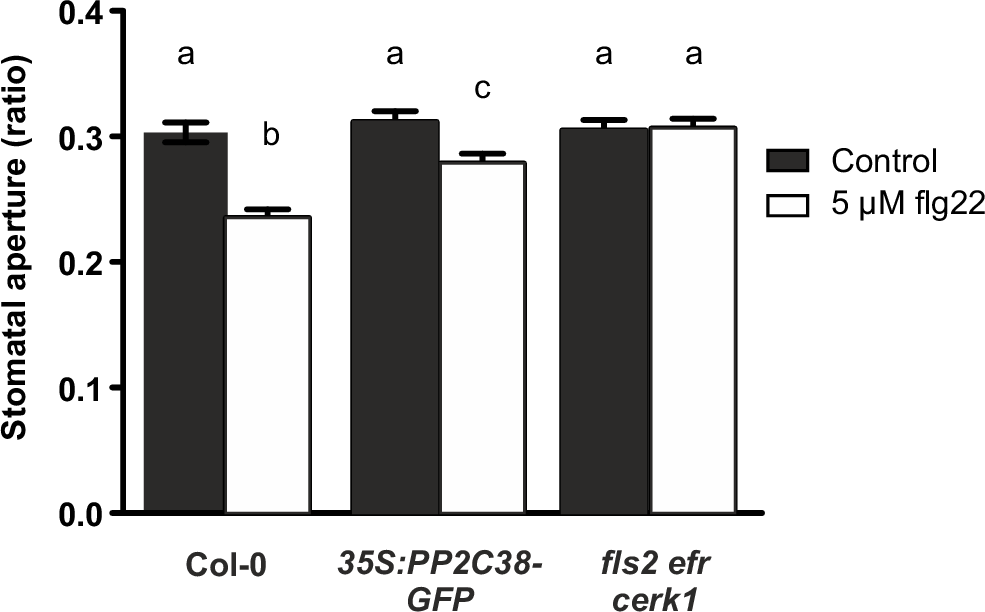

Supplement: S8 Fig — Stomatal aperture was measured 2 h after 5 μM flg22. Values are mean ± SE (n>60; one-way ANOVA, Tukey post hoc test). Different letters indicate significantly different values at p < 0.001. Experiment replicated three times with similar results. (TIF) [file ppat.1005811.s008.tif]

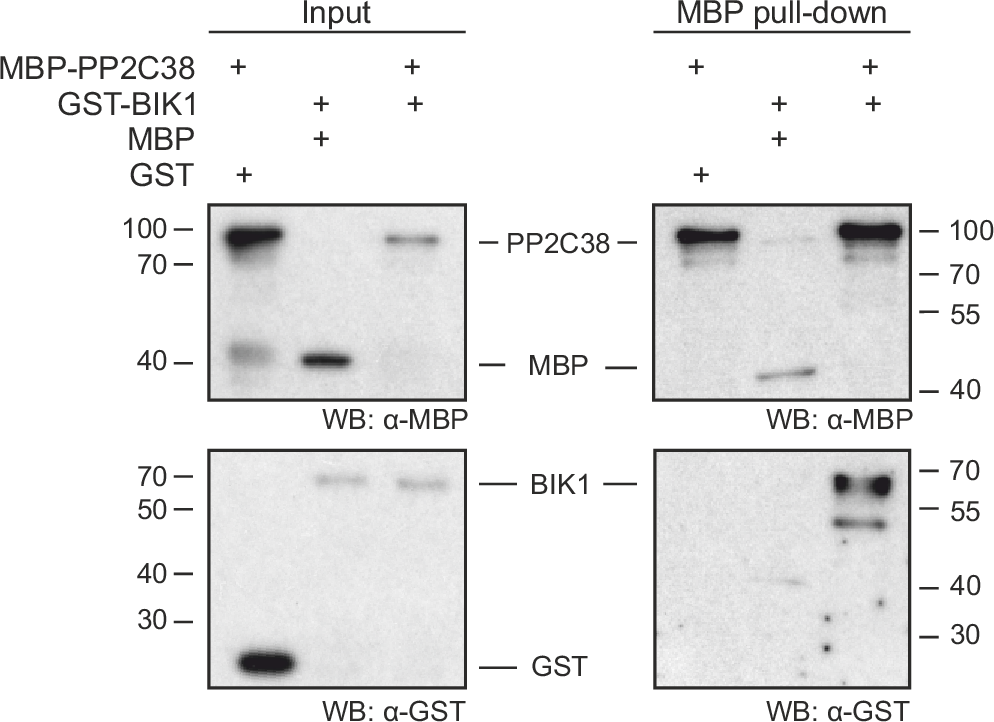

Supplement: S9 Fig — In vitro MBP pull-down performed with recombinant MBP-PP2C38 and GST-BIK1. Free MBP and GST proteins used as control. Experiment repeated three times with similar results. (TIF) [file ppat.1005811.s009.tif]

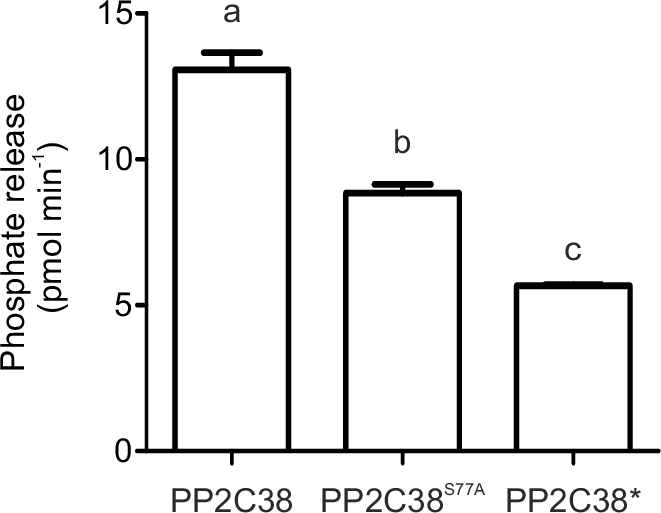

Supplement: S10 Fig — Recombinant MBP-PP2C38, MBP-PP2C38S77A or MBP-PP2C38* proteins were incubated with a synthetic phosphopeptide in the presence or absence of Mg2+ ions. Release of inorganic phosphate was quantified using a colorimetric assay. Values are averages ± SD (n = 3). Letters indicate statistically significant differences based on ANOVA, Dunnet post hoc test, p < 0.001. (TIF) [file ppat.1005811.s010.tif]

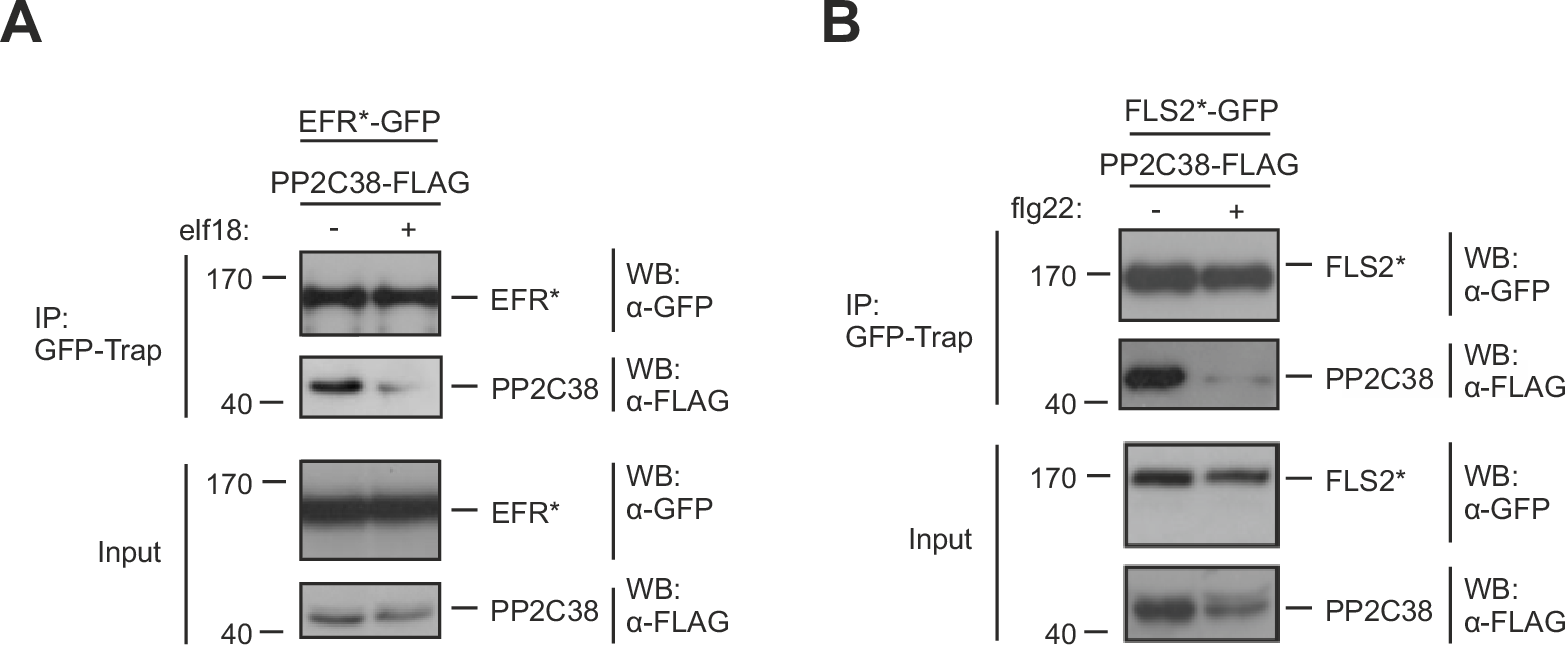

Supplement: S11 Fig — (A-B) PP2C38 and kinase-dead versions of EFR* (A) or FLS2* (B) proteins were co-immunoprecipitated from N. benthamiana leaves treated (+) or not (-) with 100 nM elf18 (A) or flg22 (B) for 20 min. (TIF) [file ppat.1005811.s011.tif]

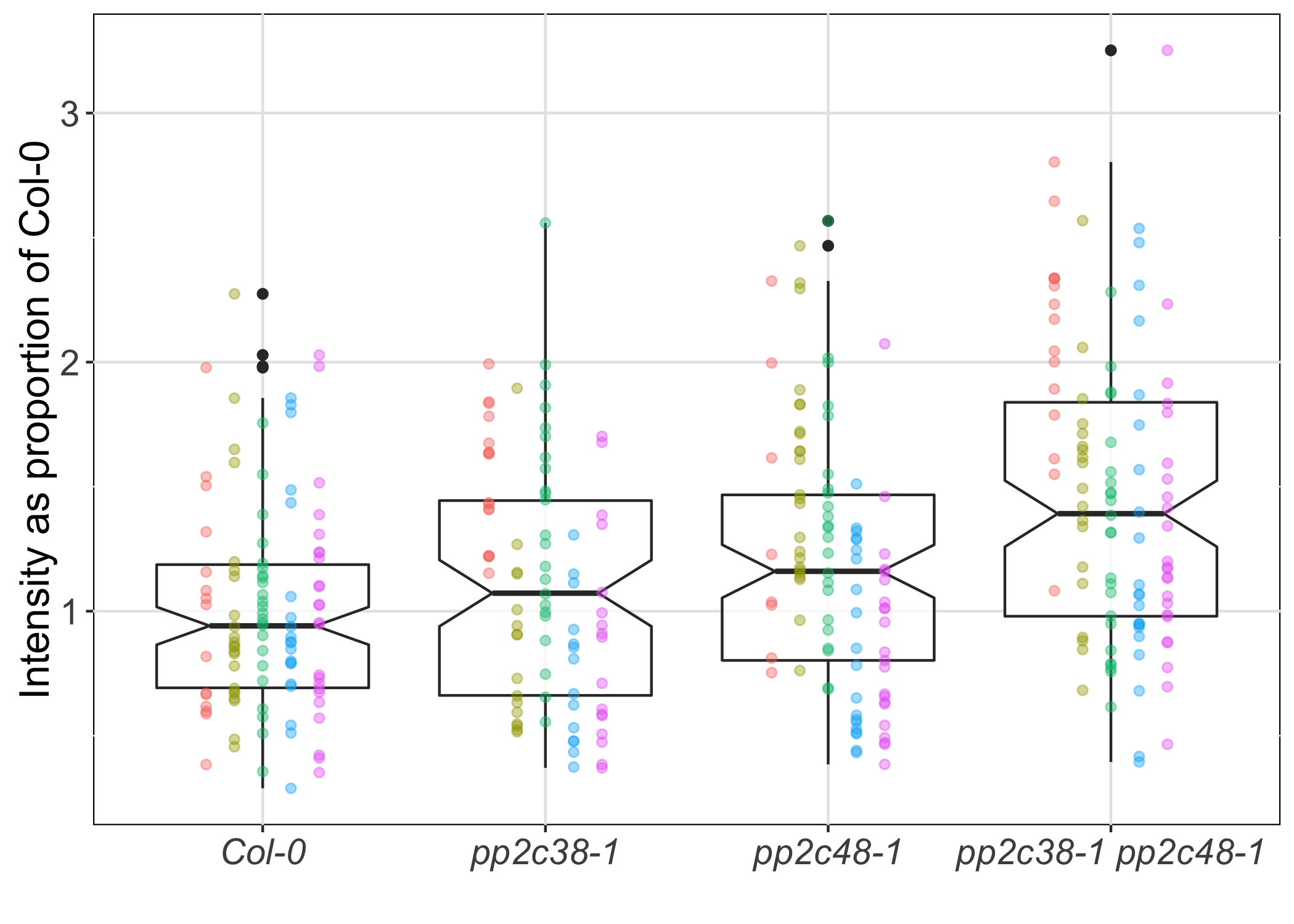

Supplement: S1 Appendix — (ZIP) [file ppat.1005811.s014.zip › pp2c/elf18.png]

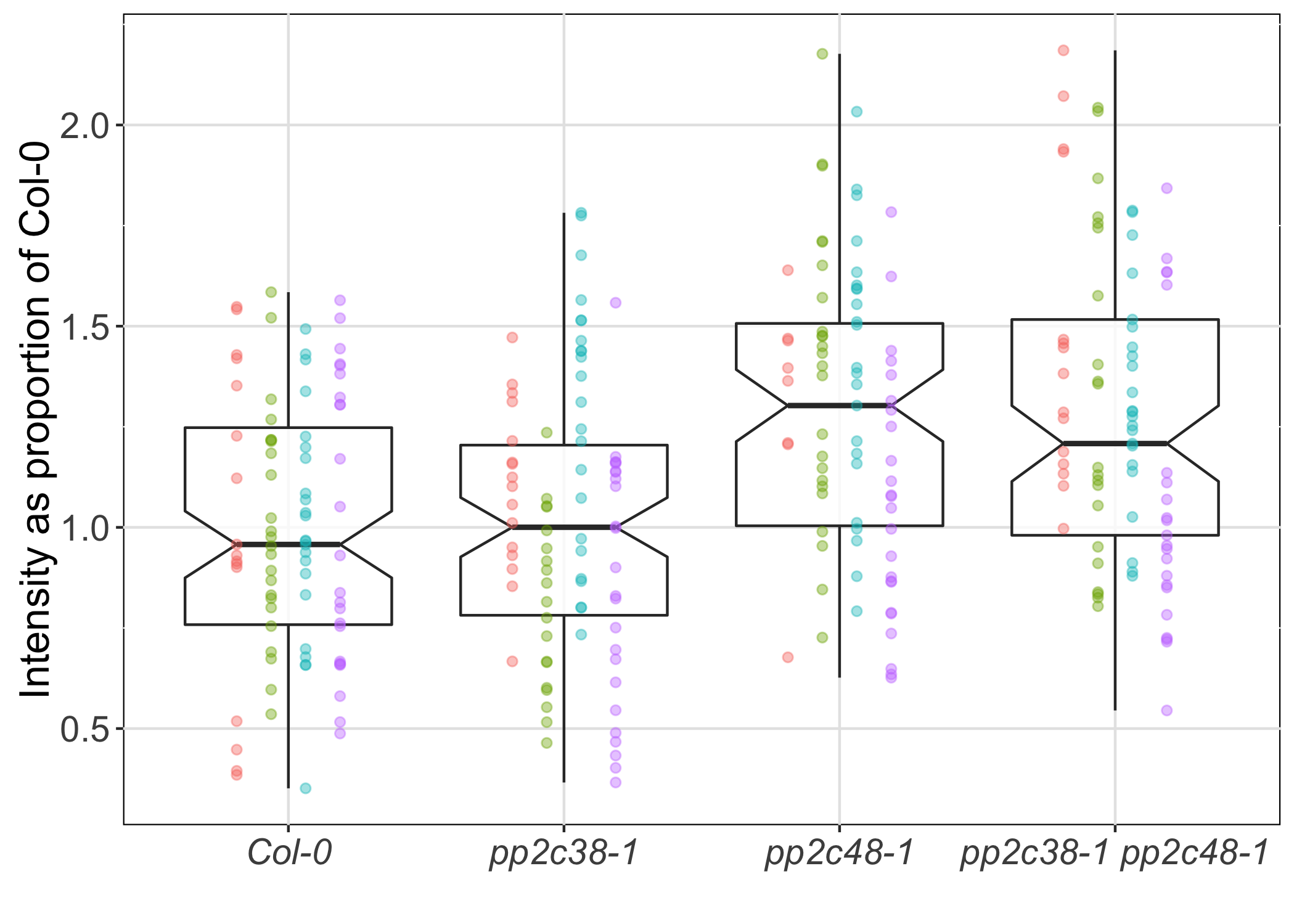

Supplement: S1 Appendix — (ZIP) [file ppat.1005811.s014.zip › pp2c/flg22.png]
