# Supplementary material for: The Arabidopsis Protein Phosphatase PP2C38 Negatively Regulates the Central Immune Kinase BIK1
Source: PLoS Pathog. 2016 Aug 5;12(8):e1005811. doi: 10.1371/journal.ppat.1005811 (PMC4975489; doi:10.1371/journal.ppat.1005811)
Supplement: S1 Table — (PDF) [file ppat.1005811.s012.pdf]

S1 Table. List of proteins interacting with EFR-CD in Y2H screen.

AGI code	Name	Predicted function
At4g28400	PP2C58	Predicted PP2C-type phosphatase
At3g12620	PP2C38	Predicted PP2C-type phosphatase
At1g22410	-	3-deoxy-7-phosphoheptulonate synthase
At3g11773	-	Electron carrier/ protein disulfide oxidoreductase
At5g63930	-	LRR-RLK (subfamily XI)
At2g20890	THYLAKOID FORMATION1 (THF1)	Involved in vesicle-mediated formation of thylakoid membranes
At1g51760	IAA-ALANINE RESISTANT 3 (IAR3)	IAA-Ala (indole-3-acetic acid alanine)-conjugate hydrolase
At2g17560	HIGH MOBILITY GROUP B4 (HMGB4)	Assembly of nucleoprotein complexes
At4g34990	MYB DOMAIN PROTEIN 32 (MYB32)	Transcription factor
