# Supplementary material for: The Arabidopsis Protein Phosphatase PP2C38 Negatively Regulates the Central Immune Kinase BIK1
Source: PLoS Pathog. 2016 Aug 5;12(8):e1005811. doi: 10.1371/journal.ppat.1005811 (PMC4975489; doi:10.1371/journal.ppat.1005811)
Supplement: S2 Table — (PDF) [file ppat.1005811.s013.pdf]

S2 Table. Primers used in this study

Primer name	Primer sequence (5'-3')
**Genotyping *pp2c38-1* and *pp2c48-1* mutant lines**	
SALK_036920L	CCTCTTCGACAACATCAGGAG
SALK_036920R	TTGCTGCCTCTCTTAGAGCTG
SALKseq_061058L	GGTATTGGAGAAGATTCTAGTCCTG
SALKseq_061058R	GACAGAGATCGGAGTTCGAGTAGC
**Quantitative RT-PCR**	
PP2C38 F1	TGTTGGAGCTTGTTGTCTGG
PP2C38 R1	ACGATAACTGAACGGCCTTG
PP2C48 F1	AGGCTGCTCGGTTTGTAAAC
PP2C48 R1	TCCTCCTCTGTTGCTACAAACC
UBQ10 F1	TGCGCTGCCAGATAATACACTATT
UBQ10 R1	TGCTGCCCAACATCAGGTT
**Molecular cloning and site-directed mutagenesis**	
EFR_CD F1	ACAACAATGCCAGTGAT GGT
EFR_CD R1	GCTACATAGTATGCATGTC
PP2C38_BamHI F1	TCAggatccGTATCATCGGCAACTATATTGCG
PP2C38_XhoI R1	CCTctcgagTCAAGTAGAAGGTCCAGC
PP2C38 GTW F1	CACCGCCAACTTGTTTATTTA
PP2C38 GTW R1	CACCATGGTATCATCGGCAAC

PP2C38_S77A F1	CTGTTAGTATGTTTGATgCTGGTCCTCAAGCTAC
PP2C38_S77A R1	GTAGCTTGAGGACCAGcATCAAACATACTAACAG
PP2C38_D87N F1	CTTTTGTTGGTGTTTATaaTGGTCATGGTGGTCC
PP2C38_D87N R1	GGACCACCATGACCAttATAAACACCAACAAAAG
PP2C38_D289N F1	GTTTCTTATATTTGCATCAaaCGGCTTGTGGGAGCAC
PP2C38_D289N R1	GTGCTCCCACAAGCCGttTGATGCAAATATAAGAAAC
PP2C58 GTW F1	CACCCATCCACAGAAGACAGTAAAAGC
PP2C58 GTW R1	CACCATGGCAGGCAGTAATATTCTCC
