# Supplementary material for: The Arabidopsis Protein Phosphatase PP2C38 Negatively Regulates the Central Immune Kinase BIK1
Source: PLoS Pathog. 2016 Aug 5;12(8):e1005811. doi: 10.1371/journal.ppat.1005811 (PMC4975489; doi:10.1371/journal.ppat.1005811)
Supplement: S1 Appendix — (ZIP) [file ppat.1005811.s014.zip › pp2c/ros_analysis_elf18.html]

ROS Counting elf18


# ROS Counting elf18

#### *Dan MacLean*

#### *25 May 2016*

We’ll begin by loading in the data and doing a quick test for normal distributions of variance. This first plot is the data grouped by experiment date and line.

```
## 
## Attaching package: 'dplyr'
```

```
## The following objects are masked from 'package:stats':
## 
##     filter, lag
```

```
## The following objects are masked from 'package:base':
## 
##     intersect, setdiff, setequal, union
```

```
## 
## Attaching package: 'reshape'
```

```
## The following object is masked from 'package:dplyr':
## 
##     rename
```

```
data <- read.csv('cleaned/ros_data_elf18.csv', header=TRUE)
basic <- ggplot(data, aes(line,intensity))
scatter <- basic + geom_jitter(aes(colour=date),position = position_dodge(width=0.5)) + theme(axis.text.x = element_text(angle = 90, hjust = 1))
scatter
```

## Initial checks

Now lets do the quick QQ plot, if everything is more or less on the diagonal then we have a rough normal distribution of variance (ie no evidence of technical experimental bias in the measurements themselves).

They look ok. Not amazing and it may bear further investigation to work out right down to a fine level, but ok for now.

## Scaling by the internal control

Now let’s just scale every reading by the internal control - the Col0 intensity for each experiment. Every intensity reading will be scaled by the arithmetic mean of the Col0 intensity readings taken on the same day. Then we’ll repeat the plots.

```
find_columbia_average <- function(x,y){
  df <- data.frame(line = x, intensity = y)
  col <- df %>% filter(line == "Col" ) 
  col_mean <- mean(col$intensity)
  df$scale <- col_mean
  return(df$scale)
}

data <- data %>%
    group_by(date) %>%
    mutate(scale_factor = find_columbia_average(line,intensity)) %>%
    mutate(scaled_intensity = intensity / scale_factor)

scaled_basic <- ggplot(data, aes(line,scaled_intensity))
scaled_scatter <- scaled_basic + geom_jitter(aes(colour=date),position = position_dodge(width=0.5)) + theme(axis.text.x = element_text(angle = 90, hjust = 1))
scaled_scatter
```

Replotting is fine, there are some overall trends already `pp2c38_pp2c48` and `pp2c48` are looking higher than `Col`

## Significance

Now do the mixed effect model - this should be able to handle some real world issues in the dataset - e.g the experimental design is missing a few technical readings in some of the replicates, so it isn’t completely balanced. We’ll apply a multiple hypothesis correction too.

```
bioreps <- cast(data, line~date, mean)
```

```
## Using scaled_intensity as value column.  Use the value argument to cast to override this choice
```

```
bioreps <- melt(bioreps)
library(nlme)
```

```
## 
## Attaching package: 'nlme'
```

```
## The following object is masked from 'package:dplyr':
## 
##     collapse
```

```
date = factor(bioreps$data)
lme.1 = lme(value ~ line, random =~ 1|date, data = bioreps)
summary(lme.1)$tTable
```

```
##                        Value Std.Error DF   t-value      p-value
## (Intercept)       1.00000000 0.1250018 12 7.9998866 3.760444e-06
## linepp2c38        0.08155518 0.1419316 12 0.5746091 5.761697e-01
## linepp2c38_pp2c48 0.49103938 0.1419316 12 3.4596906 4.719847e-03
## linepp2c48        0.19910992 0.1419316 12 1.4028584 1.859991e-01
```

```
p.adjust(summary(lme.1)$tTable[-1,'p-value'])
```

```
##        linepp2c38 linepp2c38_pp2c48        linepp2c48 
##        0.57616973        0.01415954        0.37199810
```

## Final Output

And now a final plot summarising the data.

```
final <- scaled_basic + geom_boxplot(notch=TRUE,alpha = 0.8 )+ geom_jitter(aes(colour=date),alpha = 0.4,position = position_dodge(width=0.5)) + theme(
  axis.text = element_text(size = 14),
  axis.text.x = element_text(face="italic"),
  axis.title = element_text(size = 16, color = "black"),
  legend.position = "none",
    panel.background = element_rect(fill = "white", color="black"),
  axis.line = element_line(colour = "black", size = 2),
  panel.grid.major = element_line(colour = "grey90")
  ) + labs(
  x = "",
  y = "Intensity as proportion of Col-0"
  ) + scale_x_discrete(labels = c("Col-0","pp2c38-1","pp2c48-1","pp2c38-1 pp2c48-1"), limits=c("Col", "pp2c38", "pp2c48", "pp2c38_pp2c48"))
ggsave("elf18.svg")
```

```
## Saving 7 x 5 in image
```

```
ggsave("elf18.png")
```

```
## Saving 7 x 5 in image
```

```
final
```
