# Supplementary material for: The Arabidopsis Protein Phosphatase PP2C38 Negatively Regulates the Central Immune Kinase BIK1
Source: PLoS Pathog. 2016 Aug 5;12(8):e1005811. doi: 10.1371/journal.ppat.1005811 (PMC4975489; doi:10.1371/journal.ppat.1005811)
Supplement: S1 Appendix — (ZIP) [file ppat.1005811.s014.zip › pp2c/ros_analysis_flg22.html]

ROS Counting flg22


# ROS Counting flg22

#### *Dan MacLean*

#### *25 May 2016*

We’ll begin by loading in the data and doing a quick test for normal distributions of variance. This first plot is the data grouped by experiment date and line.

```
## 
## Attaching package: 'dplyr'
```

```
## The following objects are masked from 'package:stats':
## 
##     filter, lag
```

```
## The following objects are masked from 'package:base':
## 
##     intersect, setdiff, setequal, union
```

```
## 
## Attaching package: 'reshape'
```

```
## The following object is masked from 'package:dplyr':
## 
##     rename
```

```
data <- read.csv('cleaned/ros_data_flg22.csv', header=TRUE)
basic <- ggplot(data, aes(line,intensity))
scatter <- basic + geom_jitter(aes(colour=date),position = position_dodge(width=0.5)) + theme(axis.text.x = element_text(angle = 90, hjust = 1))
scatter
```

## Initial checks

Now lets do the quick QQ plot, if everything is more or less on the diagonal then we have a rough normal distribution of variance (ie no evidence of technical experimental bias in the measurements themselves).

## Scaling by the internal control

Now let’s just scale every reading by the internal control - the Col0 intensity for each experiment. Every intensity reading will be scaled by the arithmetic mean of the Col0 intensity readings taken on the same day. Then we’ll repeat the plots.

```
find_columbia_average <- function(x,y){
  df <- data.frame(line = x, intensity = y)
  col <- df %>% filter(line == "Col" ) 
  col_mean <- mean(col$intensity)
  df$scale <- col_mean
  return(df$scale)
}

data <- data %>%
    group_by(date) %>%
    mutate(scale_factor = find_columbia_average(line,intensity)) %>%
    mutate(scaled_intensity = intensity / scale_factor)

scaled_basic <- ggplot(data, aes(line,scaled_intensity))
scaled_scatter <- scaled_basic + geom_jitter(aes(colour=date),position = position_dodge(width=0.5)) + theme(axis.text.x = element_text(angle = 90, hjust = 1))
scaled_scatter
```

## Significance

Now do the mixed effect model - this should be able to handle some real world issues in the dataset - e.g again the experimental design is missing a few technical readings in some of the replicates, so it isn’t completely balanced. We’ll apply a multiple hypothesis correction too.

```
bioreps <- cast(data, line~date, mean)
```

```
## Using scaled_intensity as value column.  Use the value argument to cast to override this choice
```

```
bioreps <- melt(bioreps)
library(nlme)
```

```
## 
## Attaching package: 'nlme'
```

```
## The following object is masked from 'package:dplyr':
## 
##     collapse
```

```
date = factor(bioreps$data)
lme.1 = lme(value ~ line, random =~ 1|date, data = bioreps)
summary(lme.1)$tTable
```

```
##                         Value  Std.Error DF     t-value      p-value
## (Intercept)       1.000000000 0.07596999  9 13.16309246 3.488585e-07
## linepp2c38        0.007982905 0.08661662  9  0.09216367 9.285869e-01
## linepp2c38_pp2c48 0.293918512 0.08661662  9  3.39332714 7.957025e-03
## linepp2c48        0.286863841 0.08661662  9  3.31188006 9.056733e-03
```

```
p.adjust(summary(lme.1)$tTable[-1,'p-value'])
```

```
##        linepp2c38 linepp2c38_pp2c48        linepp2c48 
##        0.92858687        0.02387107        0.02387107
```

## Final Output

```
final <- scaled_basic + geom_boxplot(notch=TRUE,alpha = 0.8 )+ geom_jitter(aes(colour=date),alpha = 0.4,position = position_dodge(width=0.5)) + theme(
  axis.text = element_text(size = 14),
  axis.text.x = element_text(face="italic"),
  axis.title = element_text(size = 16, color = "black"),
  legend.position = "none",
    panel.background = element_rect(fill = "white", color="black"),
  axis.line = element_line(colour = "black", size = 2),
  panel.grid.major = element_line(colour = "grey90")
  ) + labs(
   x = "",
  y = "Intensity as proportion of Col-0"
  ) + scale_x_discrete(labels = c("Col-0","pp2c38-1","pp2c48-1","pp2c38-1 pp2c48-1"), limits=c("Col", "pp2c38", "pp2c48", "pp2c38_pp2c48"))

ggsave("flg22.svg")
```

```
## Saving 7 x 5 in image
```

```
ggsave("flg22.png")
```

```
## Saving 7 x 5 in image
```

```
final
```

```
library(nlme)
date = factor(bioreps$data)
lme.1 = lme(value ~ line, random =~ 1|date, data = bioreps)
summary(lme.1)$tTable
```

```
##                         Value  Std.Error DF     t-value      p-value
## (Intercept)       1.000000000 0.07596999  9 13.16309247 3.488585e-07
## linepp2c38        0.007982905 0.08661662  9  0.09216367 9.285869e-01
## linepp2c38_pp2c48 0.293918512 0.08661662  9  3.39332714 7.957025e-03
## linepp2c48        0.286863841 0.08661662  9  3.31188005 9.056733e-03
```

```
p.adjust(summary(lme.1)$tTable[-1,'p-value'])
```

```
##        linepp2c38 linepp2c38_pp2c48        linepp2c48 
##        0.92858687        0.02387108        0.02387108
```
